# Stepwise Stiffening Chromophore Strategy Realizes a Series of Ultralong Blue Room‐Temperature Phosphorescent Materials

**DOI:** 10.1002/advs.202402632

**Published:** 2024-06-24

**Authors:** Zhihao Guan, Zhaorun Tang, Jianwen Zeng, Yuewei Zheng, Lin Ding, Dongzhi Chen, Houbin Li, Xinghai Liu

**Affiliations:** ^1^ Hubei Engineering Technology Research Center of Spectrum and Imaging Instrument School of Electronic Information Wuhan University Wuhan 430072 P. R. China; ^2^ State Key Laboratory of New Textile Materials & Advanced Processing Technology Wuhan Textile University Wuhan 430073 P. R. China

**Keywords:** multi‐substrate adaptation, room‐temperature phosphorescent materials, stepwise stiffening chromophore strategy, ultralong lifetime

## Abstract

Ultralong room‐temperature phosphorescent (URTP) materials have attracted wide attention in anti‐counterfeiting, optoelectronic display, and bio‐imaging due to their special optical properties. However, room‐temperature blue phosphorescent materials are very scarce during applications because of the need to simultaneously populate and stabilize high‐energy excited states. In this work, a stepwise stiffening chromophore strategy is proposed to suppress non‐radiative jump by continuously reducing the internal spin of the chromophore, and successfully developing a series of blue phosphorescent materials. Phosphorescence lifetimes of more than 3 s are achieved, with the longest lifetime reaching 5.44 s and lasting more than 70 s in the naked eye. As far as is know, this is the best result that has been reported. By adjusting the chromophore conjugation, multicolor phosphorescences from cyan to green have been realized. In addition, these chromophores exhibit the same excellent optical properties in urea and polyvinyl alcohmance (PVA). Finally, these materials are successfully applied to luminescent displays.

## Graphical abstract

In this work, a stepwise stiffening chromophore strategy is proposed to suppress non‐radiative jump by continuously reducing the internal spin of the chromophore, and successfully developing a series of blue phosphorescent materials. Phosphorescence lifetimes of >3 s are achieved, with the longest lifetime reaching 5.44 s and lasting >70 s in the naked eye.

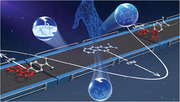

## 1 Introduction

Room‐temperature phosphorescent (RTP) materials have a wide range of applications in the fields of anti‐counterfeiting, light‐emitting displays, information transmission, and bio‐imaging due to their excellent photophysical properties.^[^ ^1^, ^2^, ^3^, ^4^, ^5^, ^6^, ^7^, ^8^, ^9^, ^10^, ^11^, ^12^, ^13^, ^14^ ^]^ Blue phosphorescence, has long been one of the main challenges in the field of phosphorescent materials, due to its need to simultaneously populate and stabilize high‐energy excited states.^[^ ^5^, ^15^, ^16^, ^17^, ^18^ ^]^ Although long‐lived room‐temperature phosphorescence many researches have been developed, the emission wavelengths of most phosphorescent materials are usually limited in long wavelength range, such as green and yellow.^[^ ^19^, ^20^, ^21^, ^22^, ^23^, ^24^ ^]^ Therefore, the development of blue URTP materials becomes very challenging.

Currently, there are two main ways to realize ultralong room temperature phosphorescence materials. The first approach is to achieve long persistent luminescence at the minute level or even at the hourly level by stabilizing the charge‐separated (CS)^[^ ^25^, ^26^, ^27^, ^28^, ^29^, ^30^ ^]^ state for long periods of time. It is worth noting that existing reports of long duration systems usually require preparation under nitrogen atmosphere to prevent quenching of the charge transfer (CT) state as well as the CS state by quenching factors such as oxygen and water. The second approach is to promote the filling of triplet excitons and stabilize them. A range of methods have been developed, including heavy atom induction,^[^ ^31^, ^32^ ^]^ small molecule crystallization,^[^ ^33^, ^34^ ^]^ polymer doping,^[^ ^35^, ^36^, ^37^, ^38^ ^]^ and hybridization frameworks.^[^ ^39^, ^40^, ^41^ ^]^ However, since the triplet excitons are easily quenched by the environment, it is difficult to realize blue room temperature phosphorescence with ultralong lifetime (>3 s). Doping the guest chromophore molecules into the rigid host matrix can effectively inhibit the nonradiative leaps of triple‐state excitons and promote the generation of long‐lived phosphorescence.^[^ ^42^, ^43^, ^44^, ^45^, ^46^, ^47^, ^48^ ^]^ Chen et al. successfully realized a blue room‐temperature phosphorescent material with a long lifetime of 1.13 s by constructing a dense hydrogen‐bonding network between the guest molecule and the host molecule, and this material was able to be stimulated by water to achieve an ultralong lifetime of 1.67 s and an ultra‐high phosphorescence quantum yield (PhQY) of 46.1%.^[^ ^44^ ^]^ Lin et al. realized a blue phosphorescent material with an ultralong lifetime of 8.74 s by doping 1,4‐phenylenediboronic acid (P2BA) into a boric acid matrix through the synergistic effect of covalent and hydrogen bonds.^[^ ^47^ ^]^ This is one of the longest lifetimes for blue room temperature phosphorescent materials. Chen et al. realized a series of multicolor, long‐lived phosphorescent materials with a maximum lifetime of 5.33 s by doping guest molecules, such as P2BA, into a urea matrix via an in situ derivatization method.^[^ ^46^ ^]^ Unfortunately, the current URTP materials are basically P2BA for the guest molecule, and the development of other URTP materials is still a great challenge.

Here, we devised a strategy of stepwise stiffening chromophore. The “rigidization” of the guest molecule itself was achieved by continuously reducing the internal rotation loss of the chromophore. By introducing the rigidized guest molecules into rigid matrix, a series of blue URTP materials were successfully realized. A series of blue URTP materials had average lifetime of up to 3 s, with naked eye afterglow durations of more than 30 s. The longest lifetimes reached 5.44 s, visible to the naked eye for more than 1 min. The chromophores can undergo conjugate expansion to achieve long‐lived cyan and yellow‐green phosphorescence emission. Excitingly, these chromophores had excellent substrate adaptability, maintaining minute naked eye durations and ultralong phosphorescence lifetimes in hydrogen‐bonding‐rich urea and PVA matrix, which will greatly expand the applications of the phosphorescent materials. Finally, these phosphorescent materials with excellent optical properties have been successfully used in anti‐counterfeit inks, aqueous media afterglow, and luminescent displays.

## 2 Result and Discussion

**Figure 1:**
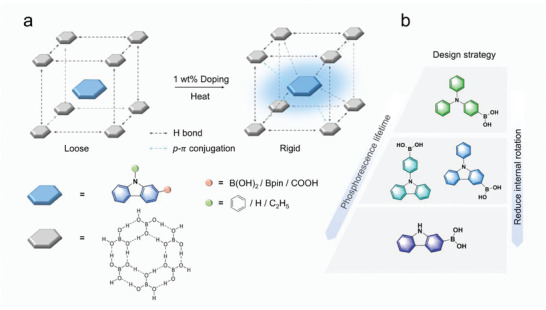
a) Preparation of URTP materials by host‐guest dual rigidity strategy. b) Breakthrough in URTP lifetime by gradual reduction of chromophore internal rotation strategy.

For the selection of guest molecule, we proposed a strategy to rigidize the chromophore by gradually reducing the internal rotation of the molecule itself, which minimized the energy loss by limiting the vibration of the chromophore itself (**Figure** **1b**). Triphenylamine (TPA) derivatives have been extensively studied due to their aggregation‐induced emission properties, among which [4‐(Diphenylamino)phenyl]boronic acid (TPBA) has a distorted molecular structure and the triplet excitons are easily dissipated by the thermal motion, resulting in relatively poor phosphorescence properties. The two benzene rings of TPBA were bridged through a C‐C bond to form the highly rigid carbazole derivative 4‐(9H‐Carbozol‐9‐yl)phenylboronicacid (4BCzB). In contrast to the twisted state of the TPBA molecule, 4BCzB have fewer rotational units. Notably, the substitution position of the boronic acid moiety can be altered to further limit the internal rotation of the chromophore itself. However, we observe that the optimized molecule 9‐Phenyl‐9H‐carbazol‐3‐ylboronic acid (3BCzB) still has units that can be internally rotated. Therefore, we removed the phenyl group and obtained a guest molecule 9H‐carbazole‐2‐boronic acid (2CzB) with a planar structural chromophore. At this point, the loss of energy is minimized, which will provide a prerequisite for ultralong‐lived triple‐state emission.

Here, we choose boric acid as the main matrix. With empty *p* orbitals and excellent ability to accept electrons, boric acid can easily form *p*‐π conjugation with the chromophore to stabilize the triplet exciton. At the same time, boric acid is rich in hydrogen bonding sites that interact with the guest molecules. After heating, boric acid can form a highly rigid network, and through various covalent/non‐covalent interactions, the guest molecules are firmly anchored in the rigid network formed, which inhibits the non‐radiative migration of the guest molecules (Figure 1a). By means of a dual rigidity strategy, we obtained a series of blue URTP materials that easily exceeded 30 s to the naked eye, with a maximum lifetime of 4.96 s. It should be mentioned that owing to the ultralong lifetime, the widely used lifetime measurement method of URTP using transient PL decay (TRES) with a flash lamp at 0.1 Hz frequency becomes not applicable. Therefore, kinetic decay was adopted here to identify the lifetime of 2CzB@BA (Figure S17b, Supporting Information). In this test mode, the phosphorescence lifetime of 2CzB@BA reached 5.44 s. This is one of the best phosphorescent performances reported (Table S14, Supporting Information).

The method of material preparation is universal. The desired phosphorescent material was obtained by mixing 1 wt% of guest molecules with boric acid, grinding it well and heating it in an oven at 100 °C for 10 min. The entire preparation process is extremely time‐consuming and the yield is almost 100%. Moreover, the method can realize the preparation of phosphorescent materials in large quantities, and a single preparation can easily reach 50 g (Figure S1, Supporting Information).

**Figure 2:**
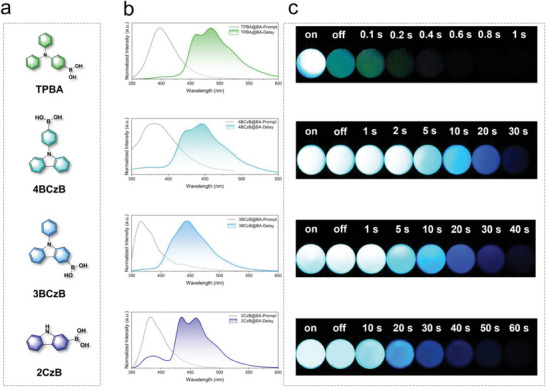
a) Guest molecules, including TPBA, 4BCzB, 3BCzB, 2CzB. b) Steady‐state and delayed spectra of TPBA@BA, 4BCzB@BA, 3BCzB@BA, and 2CzB@BA (delay time 10 ms). c) The afterglow photos of TPBA@BA, 4BCzB@BA, 3BCzB@BA, and 2CzB@BA.

TPBA, 4BCzB, 3BCzB and 2CzB were prepared by mixing with boric acid and heating to obtain TPBA@BA, 4BCzB@BA, 3BCzB@BA and 2CzB@BA respectively. As can be seen from **Figure** **2b,c**, TPBA@BA exhibits blue intense fluorescence at 310 nm UV lamp, with steady state emission located at 397 nm. When the UV lamp was turned off, only cyan phosphorescence lasting less than 1 s was observed, and the delayed emission center is located at 485 nm. This suggests that TPBA molecules with twisted states are difficult to confined by the boric acid matrix. When the twisted state of TPBA was confined, the 4BCzB molecule was obtained. 4BCzB@BA exhibited bright blue fluorescence and ultralong‐lived blue phosphorescence. The steady state and delayed emission of 4BCz@BA are located at 381 and 446 nm, respectively, and the blue afterglow duration reaches ≈30 s after turning off the UV lamp. The non‐radiative jump of the chromophore itself was further suppressed by changing the substitution position of the boric acid group. Compared to 4BCzB@BA, the emission color of 3BCzB@BA did not change. Finally, the phenyl group of the chromophore was removed and the internal rotation of the chromophore itself is completely suppressed to obtain the guest molecule 2CzB with planar chromophores. Surprisingly, 2CzB@BA exhibited bright blue afterglow with an extraordinarily long phosphorescence duration, lasting for ≈70 s to naked eye.

**Figure 3:**
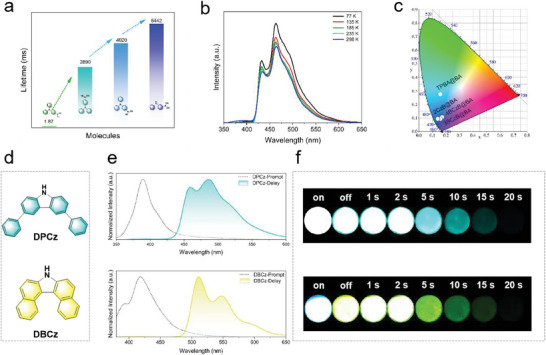
a) The phosphorescent lifetime of TPBA@BA, 4BCzB@BA, 3BCzB@BA, and 2CzB@BA. b) Variable‐temperature phosphorescence spectra of 2CzB@BA at 77, 135, 185, 235, and 298 K. c) Chromaticity coordinates (x, y) calculated from the phosphorescence spectra of TPBA@BA, 4BCzB@BA, 3BCzB@BA, and 2CzB@BA. d) Conjugate expansion molecules, including DPCz and DBCz. e) Steady‐state and delayed spectra of DPCz@BA, and DBCz@BA (delay time 10 ms). f) The afterglow photos of DPCz@BA, and DBCz@BA.

The phosphorescence lifetime of the material gradually increases as the internal rotation of the chromophore gradually decreases. The phosphorescence lifetime (τ_avg_) and quantum yield (QY) of TPBA@BA were measured (Figure S8 and Table S2, Supporting Information).The phosphorescence lifetime of TPBA@BA is only 1.87 ms, which is attributed to the significant energy dissipation caused by the twisted state of TPA in TPBA molecule. With the gradual reduction of molecular internal rotation and the promotion of molecular rigidity, the phosphorescence lifetime is gradually increased from 1.87 ms to 5.44 s, an enhancement of ≈2000 times (**Figure** **3a**). Phosphorescent intensity also increased substantially under the same test conditions (Figure S9, Supporting Information). The variable‐temperature phosphorescence spectra of 2CzB@BA were measured, and the emission peaks all showed a decreasing trend as the temperature increased from 77 K to 298 K, proving the absence of a thermally activated delayed fluorescence (TADF) component (Figure 3b). It is noteworthy that the phosphorescence emission intensity only decreased by ≈20% from 77 to 298 K, reflecting the good stability of 2CzB@BA. The delayed emission spectra of 2CzB@BA were measured for different delay times (Figure S10, Supporting Information), and the delayed spectra of 2CzB@BA remained almost unchanged as the delay time increased, with only a slight decrease in intensity occurring. CIE coordinate plots showed that URTP materials all show blue/dark blue phosphorescence (Figure 3c). Due to the rarity of this blue URTP material, we tried some similar guest molecules with the similar chromophore including (9‐phenyl‐9H‐carbazol‐2‐yl)boronic acid (2BCzB), 9‐Phenyl‐3,6‐bis(4,4,5,5‐tetramethyl‐1,3,2‐dioxaborolan‐2‐yl)−9h‐carbazole (36BCzB), 9H‐Carbazole, 3,6‐bis(4,4,5,5‐tetramethyl‐1,3,2‐dioxaborolan‐2‐yl)‐ (36CzB) and 9‐Ethylcarbazole‐3‐boronic acid (EtCzB). These molecules all have rigid carbazole‐like chromophores, and we tried to dope these guest molecules into boric acid to prepare a series of URTP materials. Excitingly, all of these URTP materials exhibited a blue URTP effect (Figure S2, Supporting Information). The photophysical properties of these URTP materials were characterized and it was found that the steady state emissions of these URTP materials were all located at 360–400 nm and showed blue‐violet/dark blue fluorescence (Figures S3–S7, Table S1, Supporting Information). The delayed emissions of these URTP materials were located at 410–450 nm and exhibited a deep blue/blue afterglow. Most of the blue URTP materials prepared had phosphorescence lifetimes in excess of 3 s. To the best of our knowledge, this is the first time that a series of URTP materials with phosphorescence lifetimes exceeding 3 s have been developed, which demonstrates the generalizability of the strategy.

In many cases, there is no way to meet the demand for URTP materials in a single color, so it is important to develop URTP materials in a variety of colors. Here, two chromophore molecules with larger structures, 3,6‐diphenyl‐9H‐carbazole (DPCz) and 7H‐dibenzo[c,g]carbazole (DBCz), were realized by chromophore conjugate expansion (Figure 3d). DPCz@BA and DBCz@BA are prepared by the same method. It is worth noting that, DPCz@BA and DPCz@BA displaying ultra long lifetime afterglow. Consistent with what we envisioned, DPCz@BA exhibits a cyan phosphorescence that lasts for >20 s to the naked eye after the UV light is turned off, while DBCz@BA exhibits a more red‐shifted yellowish‐green phosphorescence that lasts for ≈15 s to the naked eye (Figure 3f). Compared with 2CzB@BA, the delayed emission spectra and the chromaticity coordinates (x, y) of DPCz@BA and DBCz@BA also show significant redshifts (Figure 3e; Figure S11, Supporting Information), which represents the success of our strategy. In addition, DPCz@BA and DBCz@BA still exhibited ultralong phosphorescence lifetimes of 2.78 s and 1.59 s, and PhQYs of 5.0% and 1.5%, respectively (Figure S12 and Table S3, Supporting Information). The modulation of PhQY is also important in designing URTP materials. Here, we tried to regulate the QY of URTP materials by introducing more *n*‐π^*^ leaps. We doped carboxyl‐substituted carbazoles‐9H‐Carbazole‐3‐carboxylic acid (3CzCA) into boric acid matrix to prepare blue URTP materials with high QY. The delayed emission spectrum of 3CzCA@BA exhibited a deep blue afterglow with the emission centered at 438 nm (Figures S13 and S14, Supporting Information). At the same time, 3CzCA@BA exhibited an extraordinarily long phosphorescence lifetime of 2.98 s and a higher PhQY of 28.9% (Figure S13 and Table S4, Supporting Information).

**Figure 4:**
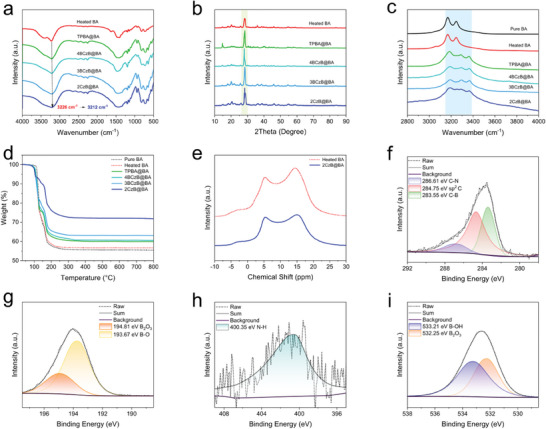
a) FT‐IR spectra of Heated BA, TPBA@BA, 4BCzB@BA, 3BCzB@BA, and 2CzB@BA. b) Powder XRD patterns of the Heated BA, TPBA@BA, 4BCzB@BA, 3BCzB@BA, and 2CzB@BA. c) Raman spectra of BA, Heated BA, TPBA@BA, 4BCzB@BA, 3BCzB@BA, and 2CzB@BA. d) TGA curves of pure BA, Heated BA, TPBA@BA, 4BCzB@BA, 3BCzB@BA, and 2CzB@BA from 30 to 800 °C. e) ^11^B NMR spectra of heated pure BA under 100°C and 2CzB@BA. High‐resolution XPS spectra of f) C 1s, g) B 1s, h) N 1s, and i) O 1s in 2CzB@BA.

To further explore the mechanism of remarkable URTP, the structure of the URTP material was analyzed through a series of characterizations. FTIR spectra (**Figure** **4a**) showed that the O‐H stretching vibration at 3226 cm^−1^ gradually broadens and shifts to the short wavelengths when the guest molecules are doped into boric acid. This is also confirmed by Raman spectroscopy (Figure 4c), where the URTP material doped with guest molecules had a significant broadening of the peak ≈3200 cm^−1^ relative to the heated pure boric acid without guest molecules, which represented the strong hydrogen bonding between the guest molecules and boric acid. In addition, the URTP material showed a significant decrease in the intensity of the B‐OH (1190 cm^−1^) and an increase in the intensity of B‐O (1490 cm^−1^) and the B‐O‐B (1320 cm^−1^) as compared to the unheated pure boric acid, which represented that this boric acid underwent dehydration upon heating. The XRD patterns show that the main peak of 28.0° of pure BA is very sharp and corresponds to the (100) crystal plane, representing the boric acid as a triclinic crystal system. The heated boric acid (Figure 4b) showed several small peaks at 18.0°, 20.2° and 25.8°, which were characteristic of metaboronic acids.^[^ ^49^ ^]^ After doping into the guest molecule, the position of the peaks remained almost unchanged, but some of the peaks became sharper, which indicated that the doping of the guest molecule into the matrix may slightly affect the crystallographic orientation of the host matrix. The TGA curves (Figure 4d) showed that the weight loss ratios of URTP materials doped with guest molecules were all smaller than those of Heated BA and Pure BA as the temperature increased, which represented that this hydrogen bonding interaction can make the URTP materials more stable to some extent. Moreover, 2CzB@BA exhibited the lowest weight loss, so the abundant hydrogen bonding interactions largely stabilized the triplet exciton of the chromophore and suppress its nonradiative leaps.

In general, covalent bonding interactions were more effective in suppressing nonradiative leaps and preventing quenching of triplet excitons. In order to investigate the interactions between the host and guest of URTP materials, we performed solid‐state NMR and XPS analysis of 2CzB@BA. ^11^B NMR (Figure 4e) further confirmed the absence of covalent bond formation between the host matrix and guest molecule by distinguishing the characteristic peaks of BA corresponding to heated BA. The XPS results further confirmed this view. The total XPS energy spectrum was shown in Figure S15 (Supporting Information). Deconvolution and fitting of the XPS spectra of C 1s, B 1s, N 1s, and O 1s of 2CzB@BA. The C 1s XPS spectrum (Figure 4f) of 2CzB@BA can be fitted with three binding energies at 286.61, 284.75, and 283.55 eV corresponding to C‐N, sp^2^C, and C‐B bonds, respectively. B 1s XPS spectrum (Figure 4g) can be fitted to two binding energies at 194.81 and 193.67 eV, attributed to B_2_O_3_ and B‐O bonds. The XPS spectrum of N 1s (Figure 4h) showed a component attributed to the N‐H bond of the guest molecule (400.35 eV). The XPS profile of O 1s (Figure 4i) showed two components that can be assigned to B‐OH (533.21 eV) and B_2_O_3_ (532.25 eV). Compared to the XPS spectra of 2CzB (Figure S16, Supporting Information), it was confirmed that no new covalent bonds were created between the host matrix and the guest molecules. Therefore, the interaction between the guest molecule and the boric acid should be hydrogen bonding interaction and *p‐*π conjugation.

To further understand the generation of URTP, the phosphorescence spectra of 2CzB at low temperatures were investigated. The delay emission spectra of 2CzB ethanol solution at 77 K were essentially the same as the delay emission spectra of 2CzB@BA (Figure S17, Supporting Information), which suggested the source of the luminescence of URTP was the doped guest molecule. The effects of different guest doping ratios and heat treatment temperatures on URTP properties were investigated. As can be seen from Figure S18 (Supporting Information), the phosphorescence intensity of 2CzB@BA was maximized when the doping ratio is 1 wt.%. As the doping ratio gradually increased to 5 wt.%, the phosphorescence emission intensity gradually decreased, which indicated that guest 2CzB aggregated in the matrix. And, a similar trend existed for the phosphorescence lifetime, which reached a maximum at 1 wt.%. The intensity of phosphorescence emission from 2CzB@BA decreased when the heat treatment temperature was increased from 100 to 200 °C (Figure S20, Supporting Information). This may be due to the gradual conversion of the BA matrix to B_2_O_3_ at high temperature, resulting in a decrease in the hydrogen‐bonding immobilization of the guest molecules. When the heat treatment temperature reached 250 °C, the phosphorescence emission intensity of 2CzB@BA decreased further, which indicated the formation of more B_2_O_3_. This is further supported by FTIR spectra and XRD patterns (Figures S21 and S22, Supporting Information). In addition, the prepared URTP materials had high stability. It can be stored for more than ten days under oxygen environment, high humidity environment, high temperature, and low temperature environment, and continuous UV illumination environment, and the phosphorescence intensity decreased ≈15% and lifetime remained stable (Figure S19, Supporting Information).

**Figure 5:**
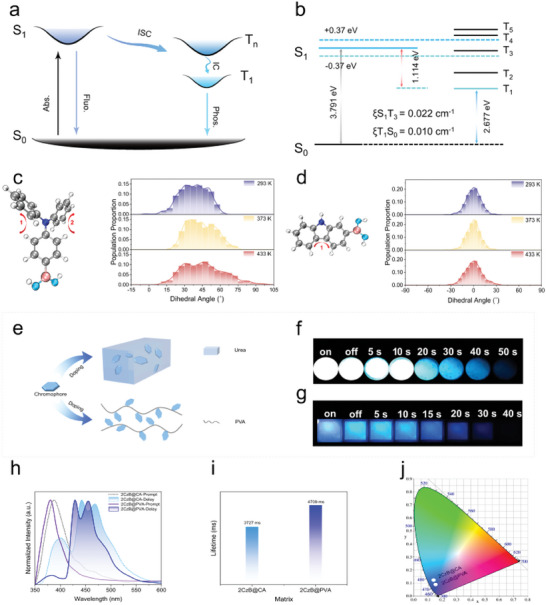
a) Phosphorescent emission mechanism of URTP materials. b) The efficient intersystem crossing channels with │*E*_S1_ *‐E*_Tn_│ < 0.37 eV and SOC constants of 2CzB@BA. c) Torsion angles of guest TPBA and the distribution of the torsion angle at position 1 of TPBA at various temperatures. d) Torsion angles of guest 2CzB and the distribution of the torsion angle at position 1 of 2CzB at various temperatures. e) Substrate expansion for generic strategies, including urea and PVA. f) The afterglow photos of 2CzB@CA. g) The afterglow photos of 2CzB@PVA. h) Steady‐state and delayed spectra of 2CzB@CA and 2CzB@PVA (delay time 10 ms). i) The phosphorescent lifetime of 2CzB@CA and 2CzB@PVA. j) Chromaticity coordinates (x, y) calculated from the phosphorescence spectra of 2CzB@CA and 2CzB@PVA.

Density functional theory (DFT) calculations further revealed the URTP mechanism. **Figure** **5a** shows the general process of phosporescence emission. Considering the role between the guest molecule and the subject, we selected the guest molecule and the BA molecule as a whole as the computational model for time‐dependent density functional theory (TD‐DFT). The natural jump orbitals (NTOs) of TPBA@BA were shown in Figure S23 (Supporting Information). For the S_1_ state, the holes were distributed on the TPA unit, while the particles were distributed on almost the entire phenyl group attached to the boronic acid, exhibiting significant charge transfer (CT) character, which promoted the inter system crossing (ISC) process from S_1_ to T_n,_^[^ ^38^ ^]^ and contributed significantly to the high QY of TPBA@BA. From TPBA@BA to 4BCzB@BA, the chromophore undergone conjugate expansion, which introduced more ^3^(π, π*) enriched features leading to enhanced phosphorescence.^[^ ^1^ ^]^ In addition the twisted to planar state transition made triplet excitons less susceptible to dissipation by thermal motion, and thus a substantial enhancement of the phosphorescence properties of 4BCzB@BA occurred. As the color‐emitting molecules gradually reduced the internal rotation from TPBA@BA to 4BCzB@BA, then to 3BCzB@BA, and finally to 2CzB@BA, the local excitation (LE) character gradually increased and the CT character gradually decreased (Figures S24–S26, Supporting Information). This made the T_1_ to S_0_ jump progressively more slow, leading to a gradual decrease in the spin‐orbit coupling (SOC) value (Figure 5b; Tables S5–S9, Supporting Information), which resulted in a longer phosphorescence lifetime.^[^ ^50^ ^]^ In order to demonstrate the effect of the rigidity of the chromophore on the phosphorescence properties, we performed molecular dynamics (MD) simulations on four guest molecules. MD calculated the distribution of torsion angles for TPBA, 4BCzB,3BCzB, and 2CzB at 293, 373, and 433 K. From Figure 5c, it can be seen that the torsion angles of TPBA had close probability distributions between 20–50° at 293 K, indicating that the phenyl group of TPBA was easy to spin close to 30° at room temperature, forming a twisted state configuration and leading to energy dissipation. As the temperature increased, the range of torsion angles became larger, reaching 10–90°, indicating that the molecular configuration of such twists was extremely unstable and highly susceptible to energy loss, which is fatal to phosphorescent properties. After linking the two spinning benzene rings, the 4BCzB molecule was obtained, at which point the spinning group became a carbazole group. It can be seen that the highest probability of torsion angle 1 was at −120° at 293 K, but its change in torsion angle fluctuation was much smaller than that of the TPBA, and the change in torsion angle 2 was very small (Figure S28, Supporting Information). After anchoring the carbazole group, the spinning group was changed to phenyl to obtain the 3BCzB molecule. As can be seen from Figure S29 (Supporting Information), the torsion angle at both positions of 3BCzB showed a smaller range of fluctuation compared to 4BCzB. Finally, the rotationally capable group was removed directly, and even with the temperature increased to 433 K, the torsion angle 1 of 2CzB remained almost constant. And the torsion angle remained at 0° to maintain the highest probability (Figure 5d). Therefore, the rigidity of the chromophore had a very strong influence on the phosphorescence properties. In our further generalized strategy design, we adjusted the conjugation of the guest molecules, it can be seen that the T_1_ energy levels of DPCz@BA and DBCz@BA showed lower energy levels (Figures S31 and S32, Tables S10 and S11, Supporting Information), which made the phosphorescence emission wavelength red‐shifted to cyan and yellow‐green colors. In addition, the replacement of the boronic acid group with a carboxyl group resulted in the introduction of more lone‐pair‐electrons leading to a significant increase in the SOC value of 3CzCA@BA^[^ ^47^ ^]^ (Figure S30 and Table S10, Supporting Information). As a result, 3CzCA@BA exhibited higher PhQY.

Till now, most studies had been focused on chromophores, but the substrate adaptation of chromophores had rarely been mentioned. Different types of matrices can broaden the application range of URTP materials to a large extent, enabling higher utilization of URTP materials in different environments. Since the guest molecules can be well anchored inside the matrix by hydrogen bonding, urea and PVA, which are rich in hydrogen bonding sites, were considered (Figure 5e). Urea‐based URTP materials were obtained by blending with urea (doping ratio of 1 wt.%) and heating at 200 °C for 30 min. Surprisingly, 2CzB@CA exhibited blue phosphorescence emission (Figure 5f). After mixing 2CzB with an aqueous solution of PVA by heating and stirring, the water was removed in an oven at 60 °C to obtain 2CzB@PVA films. 2CzB@PVA films also exhibited dark blue phosphorescence visible to the naked eye for more than 40 s (Figure 5g). Both 2CzB@CA and 2CzB@PVA exhibited extraordinarily long phosphorescence lifetimes of 3.73 s and 4.71 s (Figure 5g; Figures S37 and S48, Supporting Information), respectively, which also reflected the substrate adaptation of the guest molecules. In addition, the phosphorescence emission spectrum of 2CzB@CA is somewhat red‐shifted relative to both 2CzB@PVA and 2CzB@BA (Figure 5h), which was well visualized on the CIE color coordinates (Figure 5j; Figures S43 and S49, Supporting Information). This can be attributed to the fact that the introduction of more carbonyls with lone‐pair‐electrons leads to a more pronounced charge separation characteristic of the whole system,^[^ ^46^ ^]^ which resulted in red‐shifted emission and higher QY (Tables S13 and S15, Supporting Information). In addition, a series of guest molecules had good matrix adaptability and exhibited excellent luminescence properties in both urea and PVA matrices (Figures S33–S52, Supporting Information).

**Figure 6:**
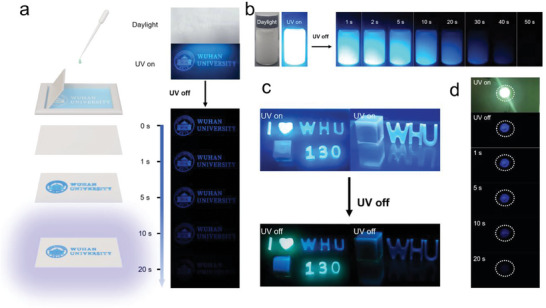
a) Patterns with URTP properties are printed on paper by screen printing methods. b) Photographs of the aqueous 2CzB@CA dispersion in daylight, under UV lamp (310 nm), and after the UV lamp was turned off. c) Fluorescence and afterglow photographs of luminescent stereo models prepared by mixing URTP material with epoxy resin. d) Photographs of an LED lamp (365 nm, 5 W) coated with URTP material after being turned on and off.

Utilizing the excellent luminescent properties of URTP materials, applications in anti‐counterfeiting and display were explored. **Figure** **6a** shows the process of preparing URTP materials into anti‐counterfeit patterns. Aqueous inks for screen printing were easily prepared by blending the guest molecules with PVA aqueous solution. The anti‐counterfeiting pattern was printed on paper by means of screen printing and dried, and the pattern can be observed by the naked eye for more than 20 s after the UV lamp was turned off. 2CzB@CA was dispersed in water, and surprisingly, a blue afterglow of ≈50 s was still observed after the UV lamp was turned off (Figure 6b). The ultralong afterglow in the aqueous medium may be due to the fact that the cyanuric acid (CA) formed by the thermal reaction of urea can well prevent water from quenching the triple state excitons of the guest molecules. And CA can form a strong hydrogen bonding network, which increased the rigidity of the system. Alternatively, a series of models in different colors and shapes can be obtained by mixing the URTP material with epoxy resin and pouring it into a mold to cool and cure (Figure 6c). Finally, the URTP material was coated on an LED lamp, and a blue afterglow of more than 20 s was observed after 365 nm LED excitation (Figure 6d).

## 3 Conclusion

In summary, we have successfully developed a series of blue room‐temperature phosphorescent materials with ultralong lifetimes through a stepwise stiffening chromophore strategy. By gradually reducing the internal rotation loss of chromophores and external rigid matrix immobilization, phosphorescent lifetime of URTP materials reached 5.44 s. A series of blue URTP materials have been developed by this strategy. To demonstrate universality of the strategy, conjugate expansion of the chromophore and change of substituents achieved URTP color modulation and QY modulation. The mechanism of URTP generation and the rationality of the modulation strategy were verified by characterization and DFT calculations. In addition, a range of guest molecules exhibited excellent URTP properties in different matrices. Finally, URTP materials were successfully used in anti‐counterfeiting and luminescent displays. This work developed a range of ultralong‐life multicolor RTP materials and provided a general strategy for designing and realizing URTP materials with excellent properties.

## 4 Experimental Section

All guest molecules as well as boric acid, urea, and PVA (Mw ≈ 80000) were purchased commercially. Deionized water (18.2 MPa) was obtained from an ultrapure water system for laboratory purposes.

### Characterization

X‐ray diffraction (XRD) was measured by a MiniFlex‐600 (Rigaku, Japan) scanned at 10° min^−1^ in the 2θ range from 10 to 90°. Fourier transform infrared spectroscopy (FTIR) spectra in the range of 4000–400 cm^−1^ were collected using a Nicolet‐5700 (Thermo Nicolet, USA). The Raman spectra in the range of 2800–4000 cm^−1^ were measured by a Confocal Raman Microscope (HORIBA Jobin Yvon, France). The X‐ray photoelectron spectroscopy (XPS) of the samples was tested using a SCIENTIFIC ESCALAB 250Xi (Thermo Fisher, USA). ^11^BNMR spectra were recorded at ambient temperature on a Bruker AVANCE NEO 400 MHz NMR spectrometer. The TGA curves were tested by synchronous thermal analyzer using Termo Gravimetry Differenfial Thermal Analyzer (Mettler‐Toledo). The samples were heated at 10 K·min^−1^ rate under N_2_ atmosphere. Prompt spectra, delay spectra, phosphorescence lifetime, and absolute photoluminescence quantum yields were measured by an FLS‐1000 (Edinburgh, UK). Photos and videos were taken with an XiaoMi‐11 cellphone.

## Conflict of Interest

The authors declare no conflict of interest.

## Supporting information

Supporting Information

## Data Availability

The data that support the findings of this study are available from the corresponding author upon reasonable request.
