# Supplementary material for: Stepwise Stiffening Chromophore Strategy Realizes a Series of Ultralong Blue Room‐Temperature Phosphorescent Materials
Source: Adv Sci (Weinh). 2024 Jun 24;11(32):2402632. doi: 10.1002/advs.202402632 (PMC11348177; doi:10.1002/advs.202402632)
Supplement: Supplementary file 1 — Supporting Information [file ADVS-11-2402632-s001.docx]

**Supporting Information**

**Stepwise Stiffening Chromophore Strategy Realizes A Series of Ultra-long Blue Room-temperature Phosphorescent Materials**

*Zhihao Guan^#^, Zhaorun Tang^#^, Jianwen Zeng, Yuewei Zheng, Lin Ding, Dongzhi Chen^*^, Houbin Li, and Xinghai Liu^*^*

*Z.Guan^1,#^, Z.Tang^1,#^, J.Zeng^1^, Y.Zheng^1^, L.Ding^1^, D.Chen^2,*^, H.Li^1^, and X.Liu^1,*^*

*^1^Hubei Engineering Technology Research Center of Spectrum and Imaging Instrument，School of Electronic Information, Wuhan University, Wuhan 430072, P. R. China*

*^2^State Key Laboratory of New Textile Materials & Advanced Processing Technology, Wuhan Textile University, Wuhan 430073, P. R. China*

**Corresponding author.*

*^#^These authors contributed equally to this work.*

*Email: chdozh_2008@163.com, liuxh@whu.edu.cn*

*
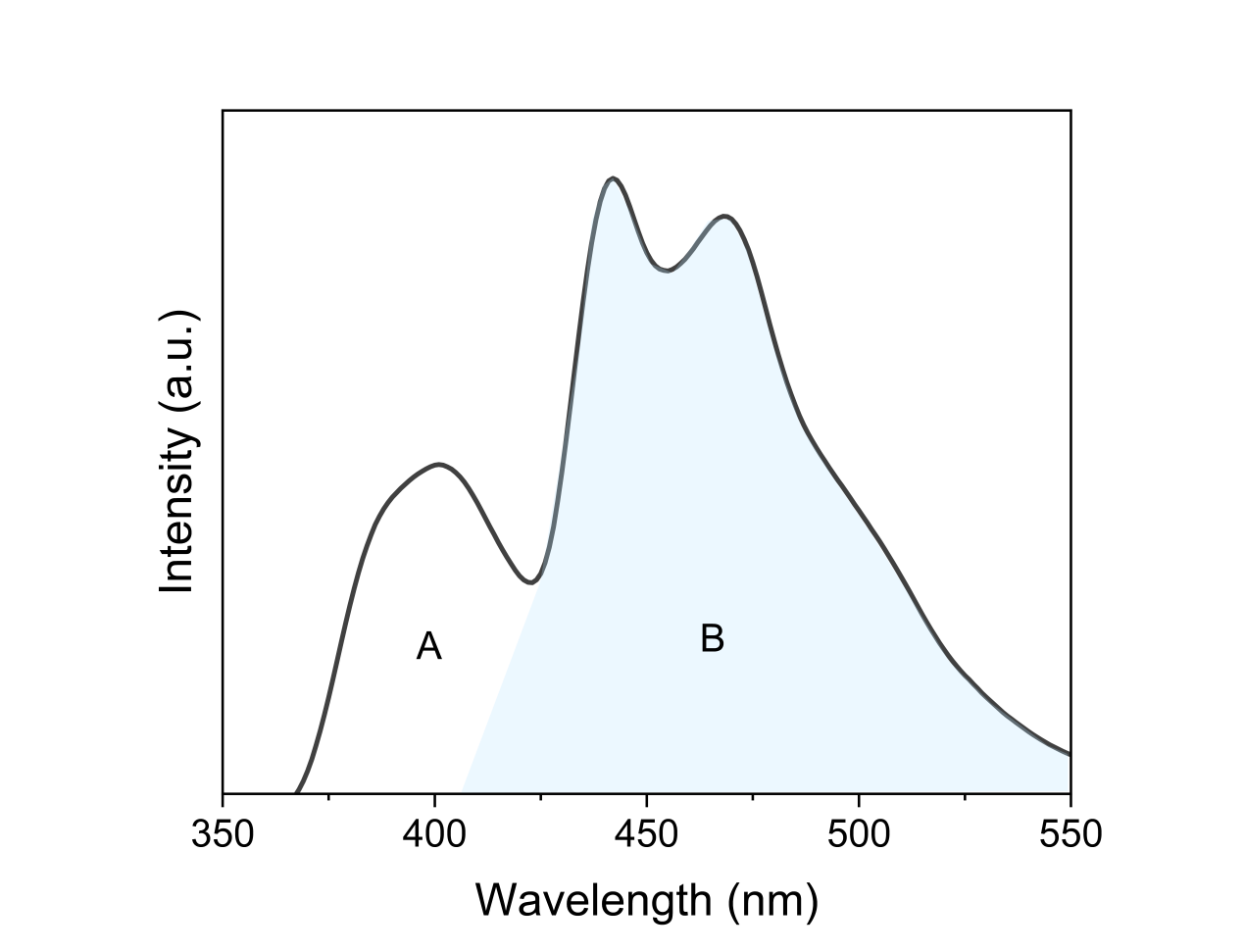
*

The absolute PLQYs of URTP materials were measured by using an Edinburgh FLS1000 spectrophotometer equipped with an integrating sphere under ambient conditions. The PhQYs of URTP materials were obtained from the following equation:

$PhQY=\frac{B}{A}\times PLQY$

where A and B represent the integral area of total photoluminescence and phosphorescence spectra, respectively. This method has been reported in previous literature ^[1]^.


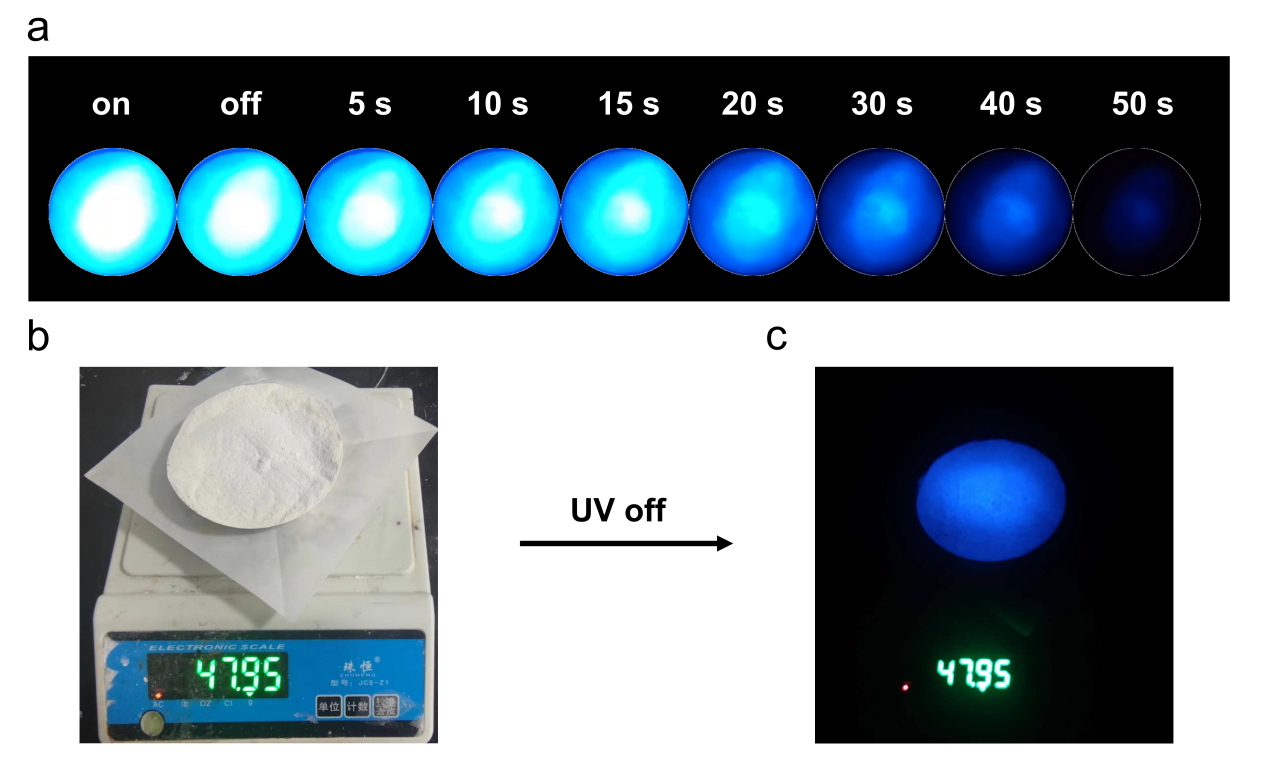


**Figure S1.** a) Afterglow photographs of bulk-prepared 2CzB@BA over time. b) The photo of 2CzB@BA in daylight. c) Afterglow photographs of 2CzB@BA in the dark.
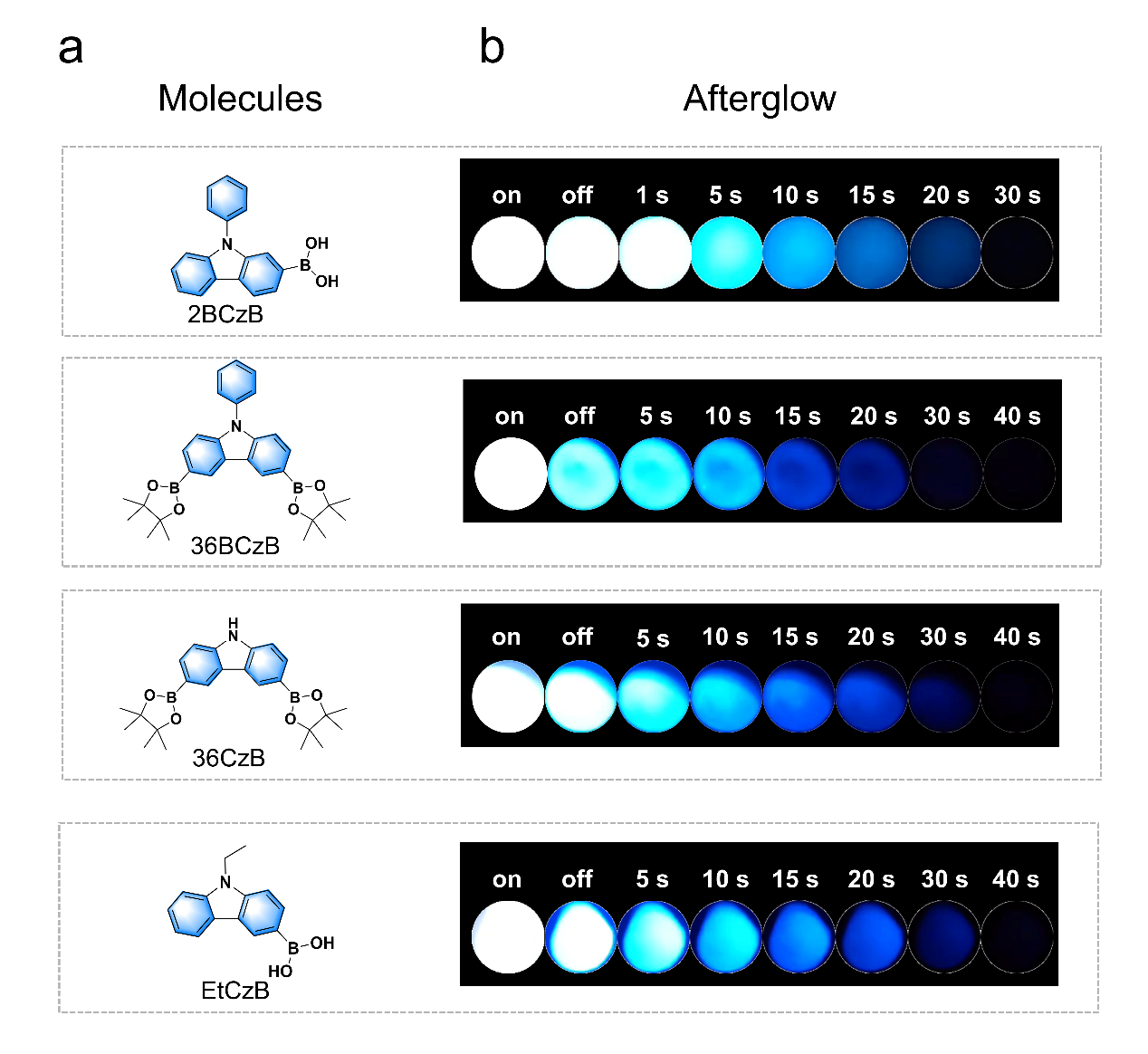


**Figure S2.** a) The structures of the guest molecules. b) The afterglow photographs of the prepared URTP materials


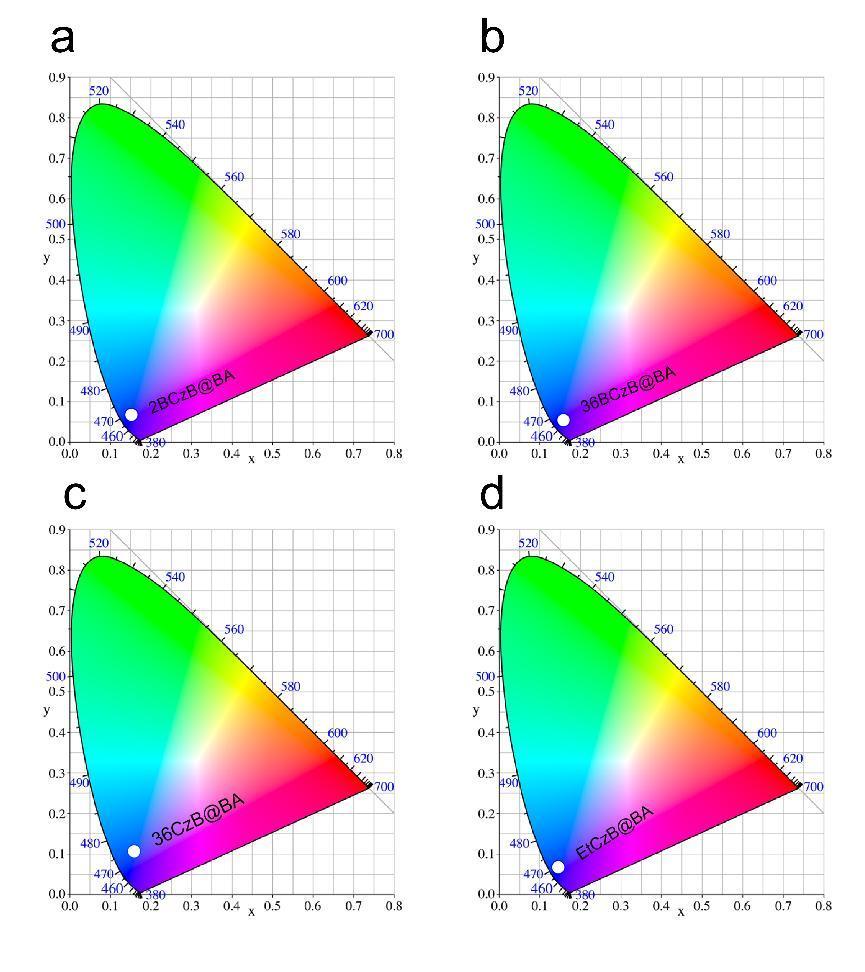


**Figure S3.** Chromaticity coordinates (x, y) calculated from the phosphorescence spectra of a) 2BCzB@BA (0.15, 0.07), b) 36BCzB@BA (0.15, 0.06), c) 36CzB@BA (0.17, 0.10), and d) EtCzB@BA (0.15, 0.07).


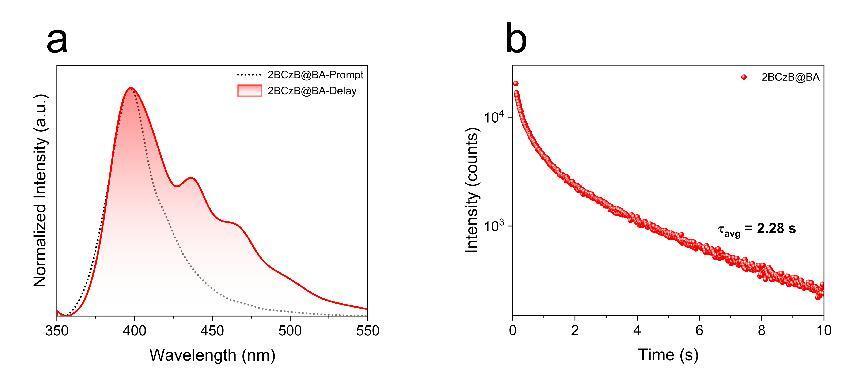


**Figure S4.** a) The prompt and delay emission spectra of 2BCzB@BA excited by 310 nm (delay time 10 ms). b) The phosphorescent lifetime of 2BCzB@BA excited by 310 nm.


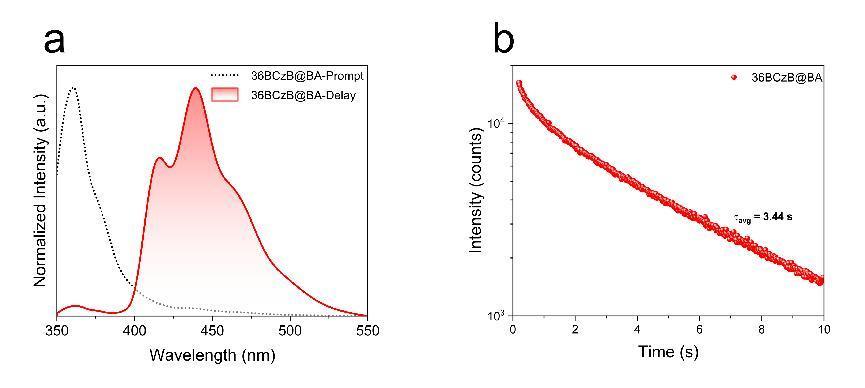


**Figure S5.** a) The prompt and delay emission spectra of 36BCzB@BA excited by 310 nm (delay time 10 ms). b) The phosphorescent lifetime of 36BCzB@BA excited by 310 nm.


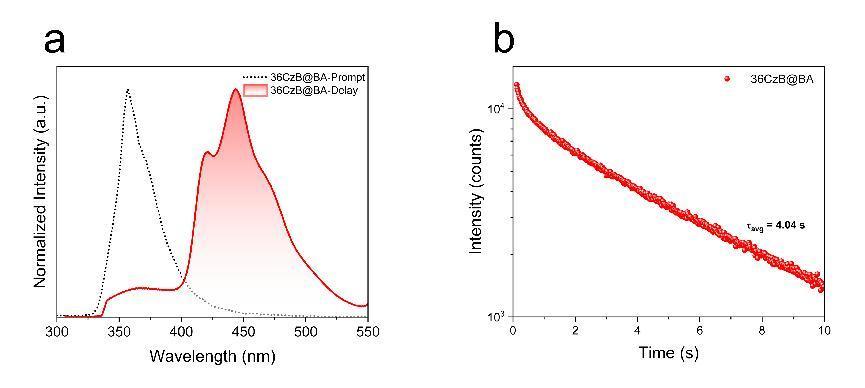


**Figure S6.** a) The prompt and delay emission spectra of 36CzB@BA excited by 310 nm (delay time 10 ms). b) The phosphorescent lifetime of 36CzB@BA excited by 310 nm.


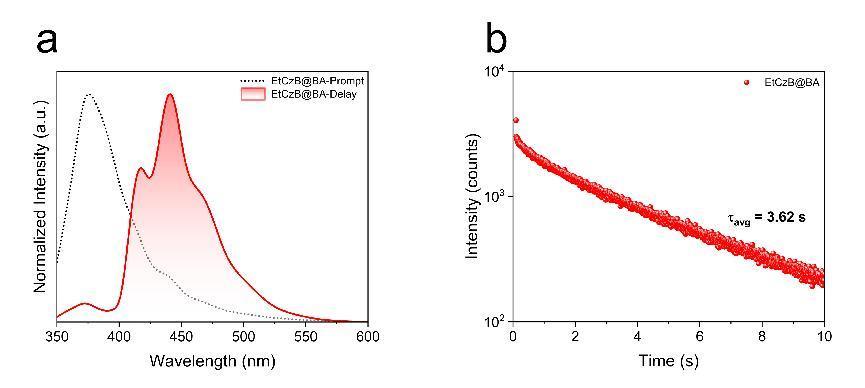


**Figure S7.** a) The prompt and delay emission spectra of EtCzB@BA excited by 310 nm (delay time 10 ms). b) The phosphorescent lifetime of EtCzB@BA excited by 310 nm.

**Table S1.** The excited wavelength (Ex), photoluminescence wavelength (PL), phosphorescent wavelength (Phos) and phosphorescent lifetime (τ_avg_) of 2BCzB@BA, 36BCzB@BA, 36CzB@BA and EtCzB@BA.

|  | Ex (nm) | PL (nm) | Phos (nm) | τ_avg_ (s) |
| --- | --- | --- | --- | --- |
| 2BCzB@BA | 310 | 397 | 437 | 2.28 |
| 36BCzB@BA | 310 | 361 | 439 | 3.44 |
| 36CzB@BA | 310 | 357 | 443 | 4.04 |
| EtCzB@BA | 310 | 375 | 441 | 3.62 |


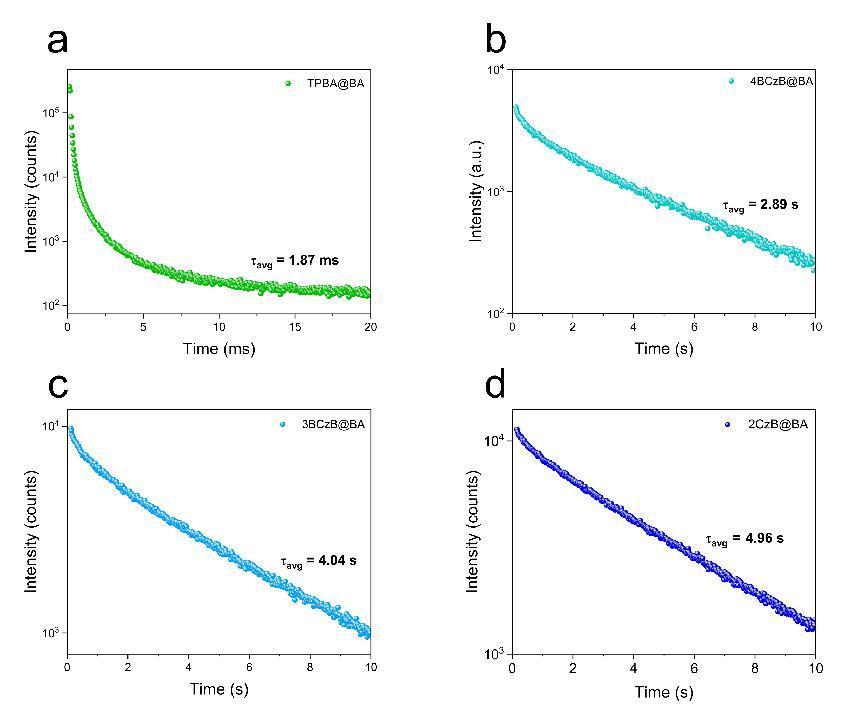


**Figure S8.** The phosphorescent lifetime of a) TPBA@BA excited by 310 nm, b) 4BCzB@BA excited by 310 nm, c) 3BCzB@BA excited by 310 nm and d) 2CzB@BA excited by 310 nm.

**Table S2.** The excited wavelength (Ex), photoluminescence wavelength (PL), phosphorescent wavelength (Phos), phosphorescent lifetime (τ_avg_), FLQY (fluorescence quantum yield), PhQY (phosphorescent quantum yield), and PhQY/PLQY of TPBA@BA, 4BCzB@BA, 3BCzB@BA and 2CzB@BA.

|  | Ex (nm) | PL (nm) | Phos (nm) | τ_avg_ (s) | FLQY | PhQY | PhQY/PLQY |
| --- | --- | --- | --- | --- | --- | --- | --- |
| TPBA@BA | 310 | 397 | 485 | 0.00187 | 42.1% | 13.9% | 23.6% |
| 4BCzB@BA | 310 | 381 | 446 | 2.89 | 22.2% | 10.8% | 32.7% |
| 3BCzB@BA | 310 | 366 | 443 | 4.04 | 18.3% | 3.7% | 16.6% |
| 2CzB@BA | 310 | 382 | 435 | 4.96 | 24.8% | 12.2% | 30.5% |


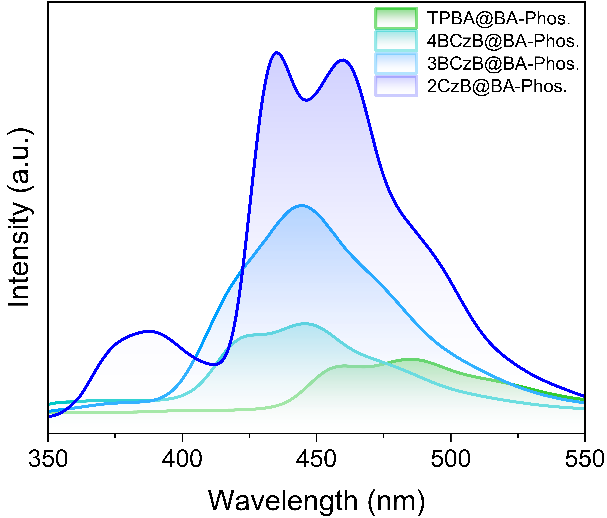


**Figure S9.** The delay emission spectra of TPBA@BA, 4BCzB@BA, 3BCzB@BA, and 2CzB@BA.


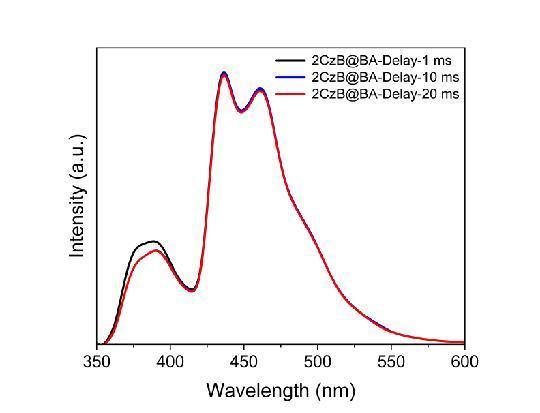


**Figure S10.** The delay emission spectra of 2CzB@BA excited by 310 nm with different delay time.


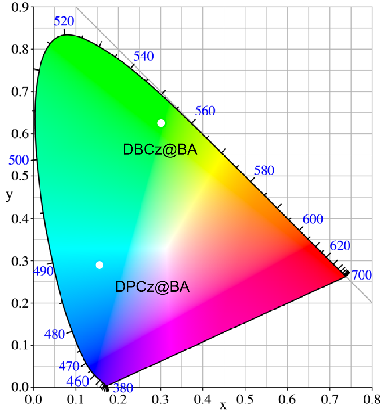


**Figure S11.** Chromaticity coordinates (x, y) calculated from the phosphorescence spectra of

DPCz@BA (0.16, 0.29) and DBCz@BA (0.31, 0.63).


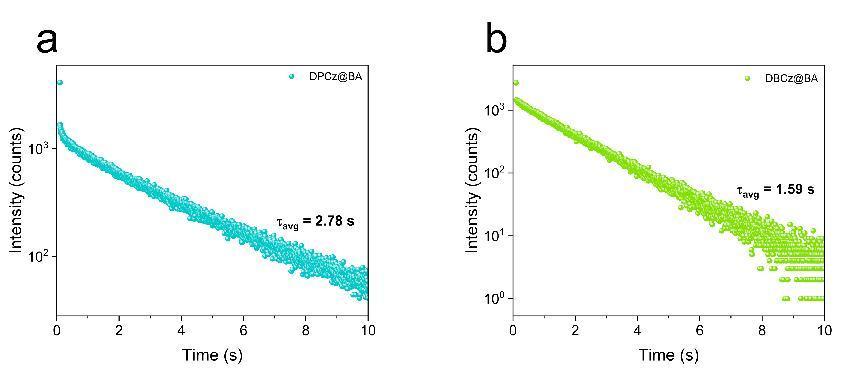


**Figure S12.** The phosphorescent lifetime of a) DPCz@BA excited by 310 nm, b) DBCz@BA excited by 360 nm.**Table S3.** The excited wavelength (Ex), photoluminescence wavelength (PL), phosphorescent wavelength (Phos), phosphorescent lifetime (τ_avg_), FLQY (fluorescence quantum yield), PhQY (phosphorescent quantum yield), and PhQY/PLQY of DPCz@BA and DBCz@BA.

|  | Ex (nm) | PL (nm) | Phos (nm) | τ_avg_ (s) | FLQY | PhQY | PhQY/PLQY |
| --- | --- | --- | --- | --- | --- | --- | --- |
| DPCz@BA | 310 | 389 | 486 | 2.78 | 28.0% | 5.0% | 15.2% |
| DBCz@BA | 360 | 418 | 512 | 1.59 | 21.5% | 1.5% | 6.5% |


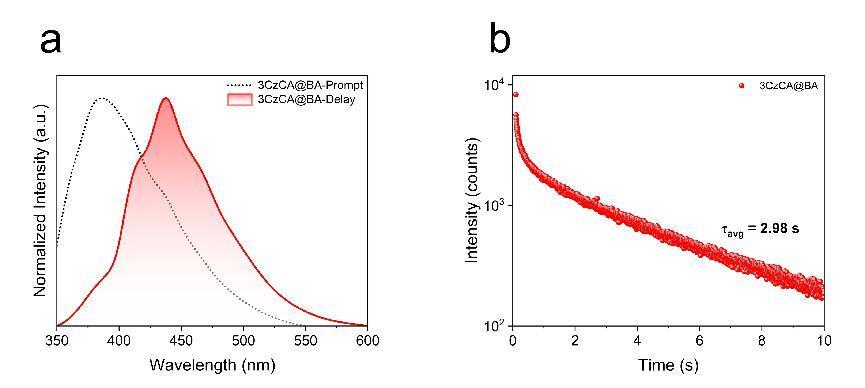


**Figure S13.** a) The prompt and delay emission spectra of 3CzCA@BA excited by 310 nm (delay time 10 ms). b) The phosphorescent lifetime of 3CzCA@BA excited by 310 nm.


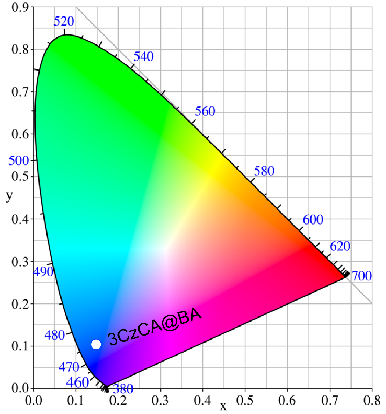


**Figure S14.** Chromaticity coordinates (x, y) calculated from the phosphorescence spectra of

3CzCA@BA (0.15, 0.10).**Table S4.** The excited wavelength (Ex), photoluminescence wavelength (PL), phosphorescent wavelength (Phos), phosphorescent lifetime (τ_avg_), FLQY (fluorescence quantum yield), PhQY (phosphorescent quantum yield), and PhQY/PLQY of 3CzCA@BA.

|  | Ex (nm) | PL (nm) | Phos (nm) | τ_avg_ (s) | FLQY | PhQY | PhQY/PLQY |
| --- | --- | --- | --- | --- | --- | --- | --- |
| 3CzCA@BA | 310 | 386 | 438 | 2.98 | 15.1% | 28.9% | 67.9% |


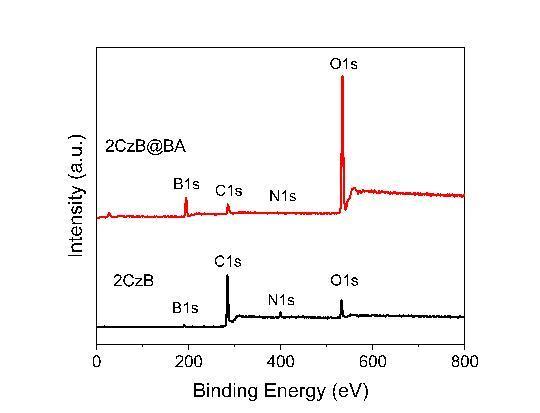


**Figure S15.** The XPS survey scan of 2CzB and 2CzB@BA.
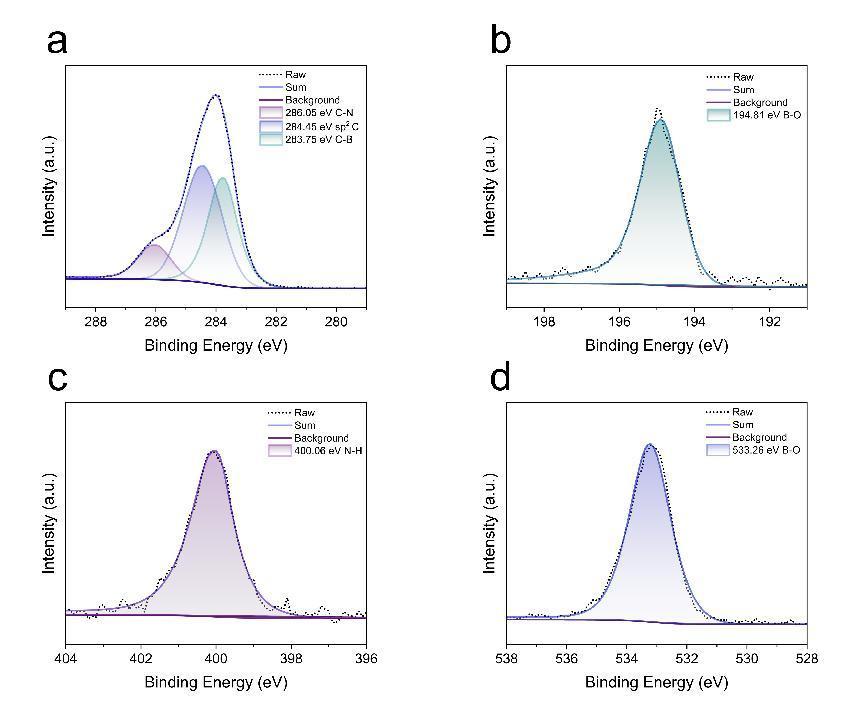


**Figure S16.** High-resolution XPS. a) C 1s, b) B 1s, c) N 1s, and d) O 1s spectra of 2CzB.


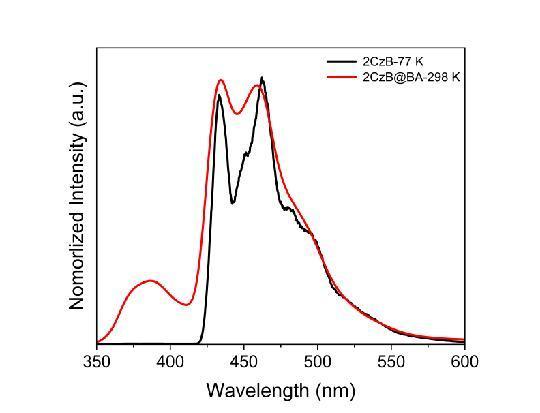


**Figure S17.** The delay emission spectra of 2CzB@BA at 298 K and 2CzB in ethanol at 77 K excited by 310 nm.


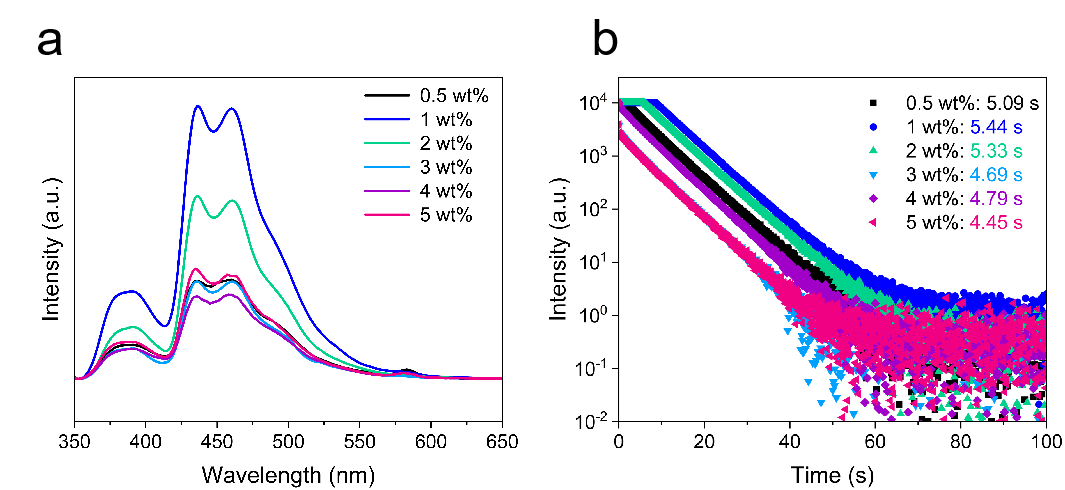


**Figure S18.** a) The delay emission spectra of 2CzB@BA with different ratios of 2CzB. b) The phosphorescent lifetime of 2CzB@BA with different ratios of 2CzB.


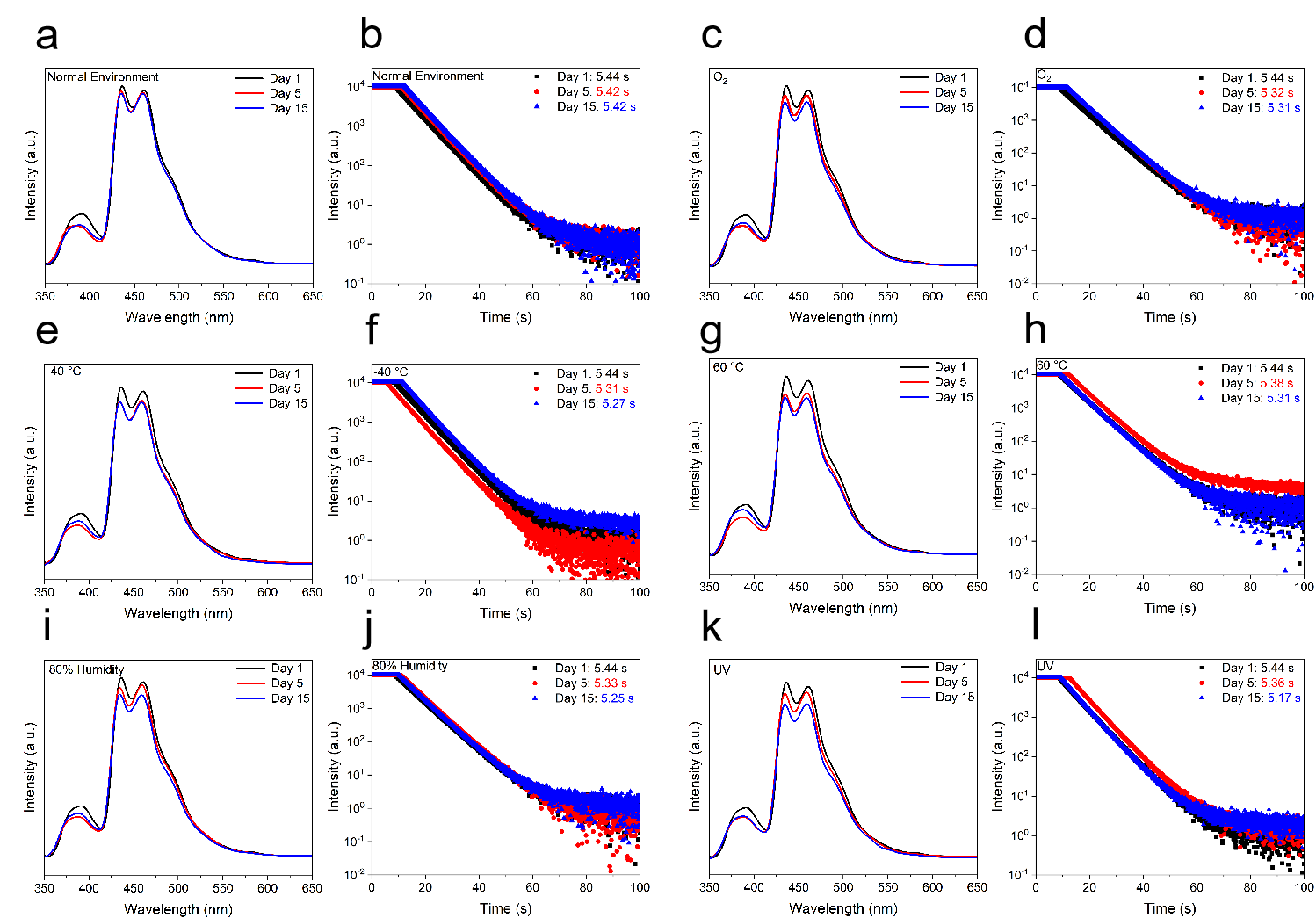


**Figure S19.** a) The delay emission spectra of 2CzB@BA at 1, 5 and 15 days in a normal storage environment. b) The phosphorescent lifetime of 2CzB@BA at 1, 5 and 15 days in a normal storage environment. c) The delay emission spectra of 2CzB@BA at 1, 5 and 15 days in an oxygen storage environment. d) The phosphorescent lifetime of 2CzB@BA at 1, 5 and 15 days in an oxygen storage environment. e) The delay emission spectra of 2CzB@BA at 1, 5 and 15 days in a storage environment of -40°C. f) The phosphorescent lifetime of 2CzB@BA at 1, 5 and 15 days in a storage environment of -40°C. g) The delay emission spectra of 2CzB@BA at 1, 5 and 15 days in a storage environment of 60°C. h) The phosphorescent lifetime of 2CzB@BA at 1, 5 and 15 days in a storage environment of 60°C. i) The delay emission spectra of 2CzB@BA at 1, 5 and 15 days in a storage environment of 80% humidity. j) The phosphorescent lifetime of 2CzB@BA at 1, 5 and 15 days in a storage environment of 80% humidity. k) The delay emission spectra of 2CzB@BA at 1, 5 and 15 days in a storage environment of constant UV illumination. l) .The phosphorescent lifetime of 2CzB@BA at 1, 5 and 15 days in a storage environment of constant UV illumination.


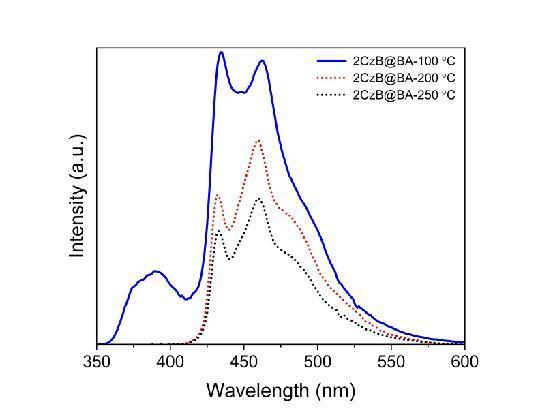


**Figure S20.** The delay emission spectra of 2CzB@BA with heat-treatment temperatures.


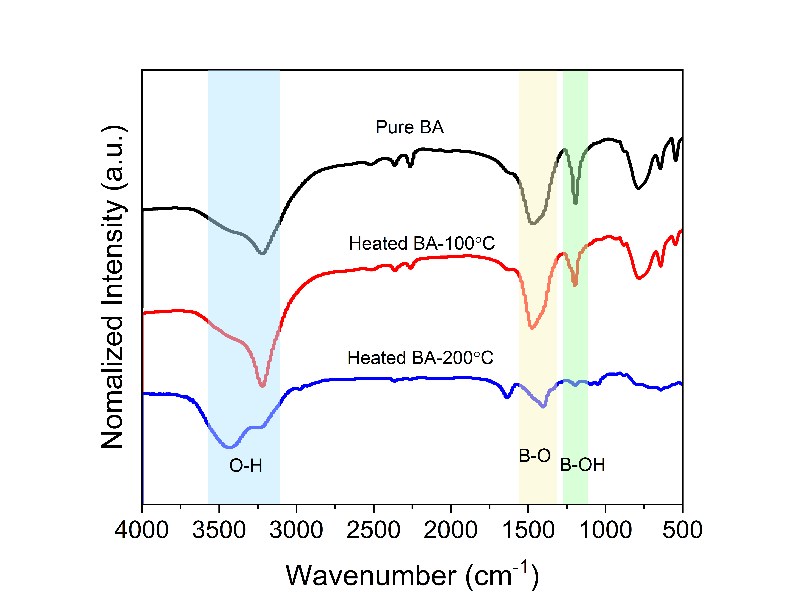


**Figure S21.** The FTIR spectra of 2CzB@BA with heat-treatment temperatures.


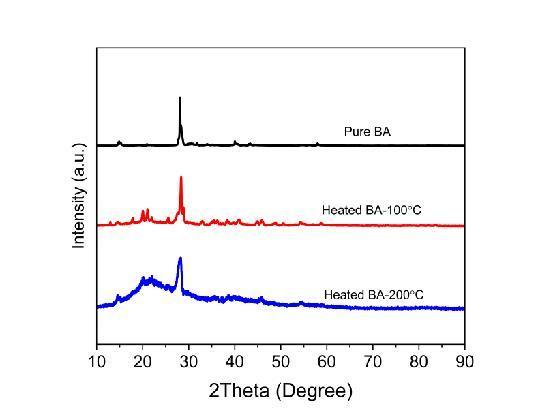


**Figure S22.** The XRD patterns of 2CzB@BA with heat-treatment temperatures.


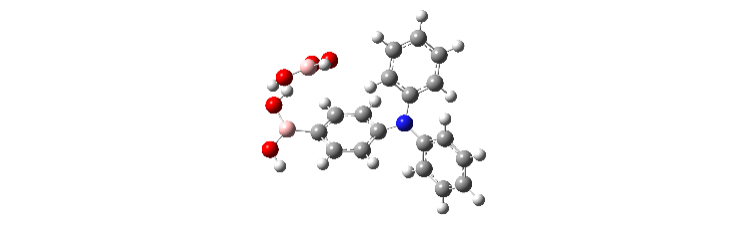


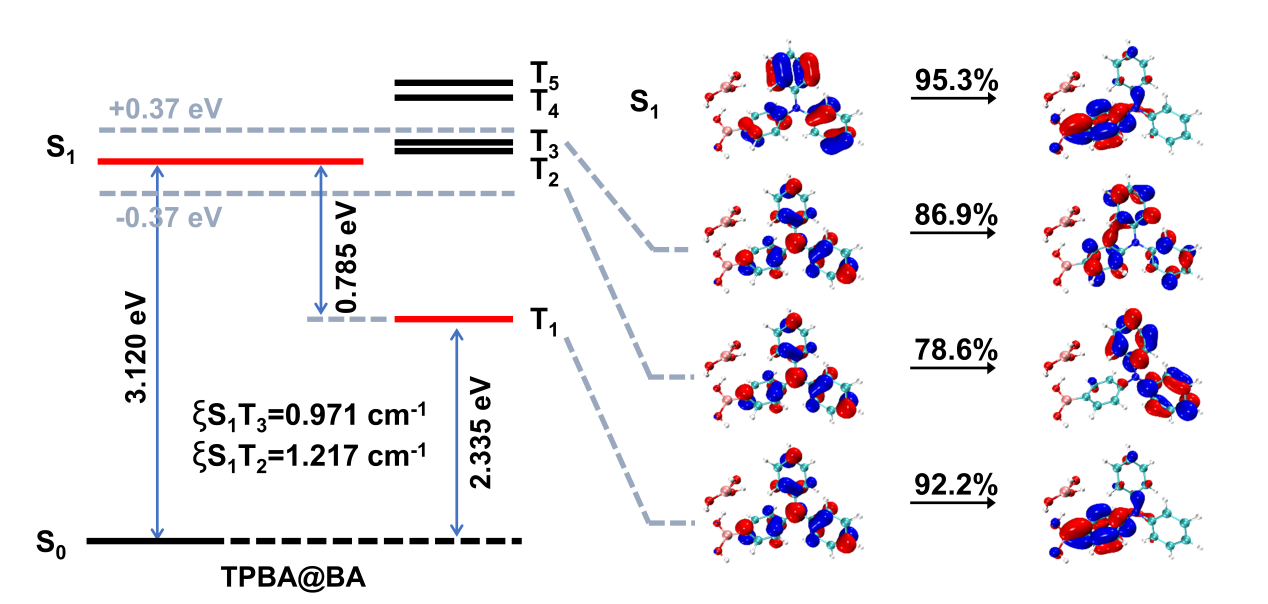


**Figure S23.** Calculated excitation energies, spin-orbit couplings (ξ) and natural transition orbitals for TPBA@BA.


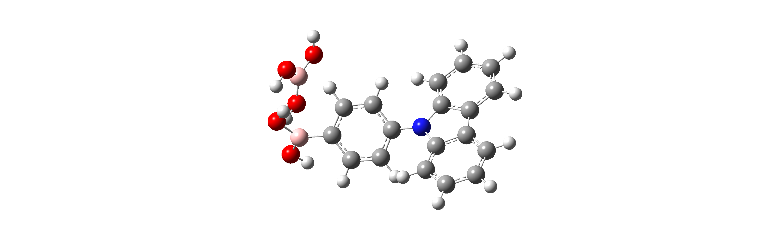


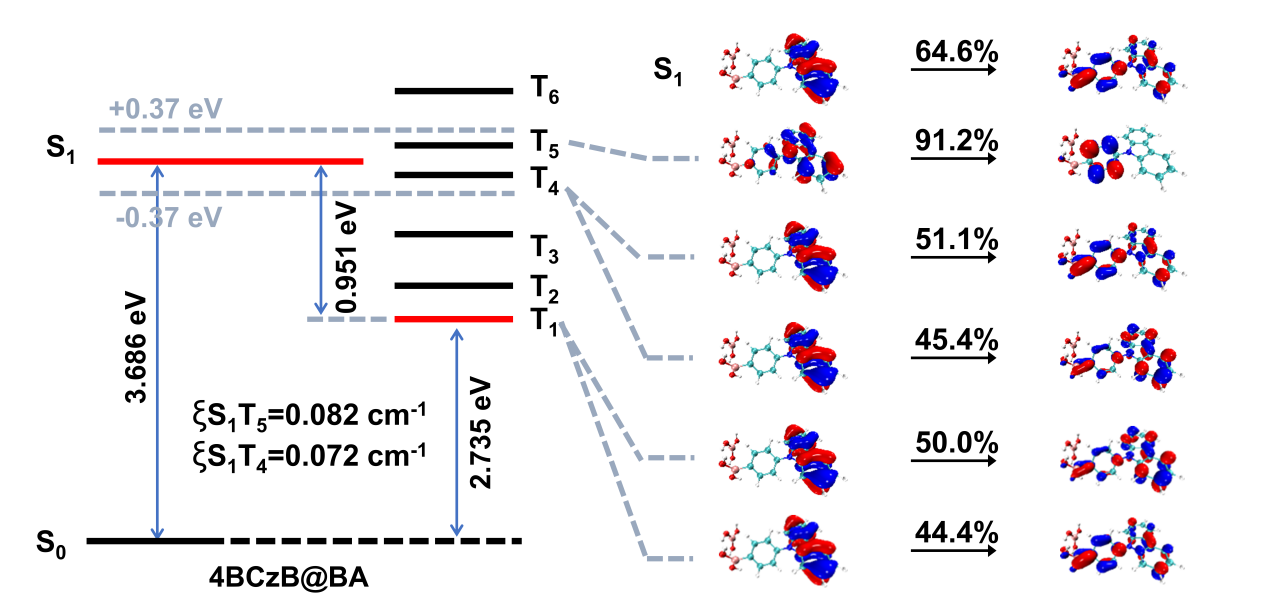


**Figure S24.** Calculated excitation energies, spin-orbit couplings (ξ) and natural transition orbitals for 4CzB@BA.


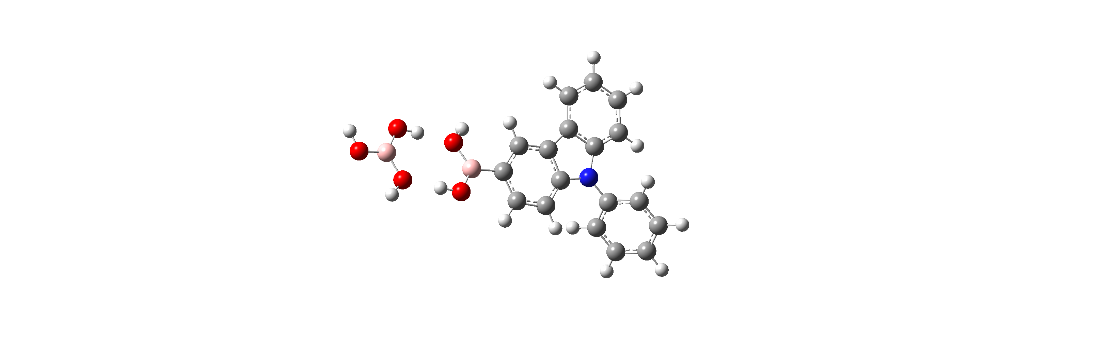


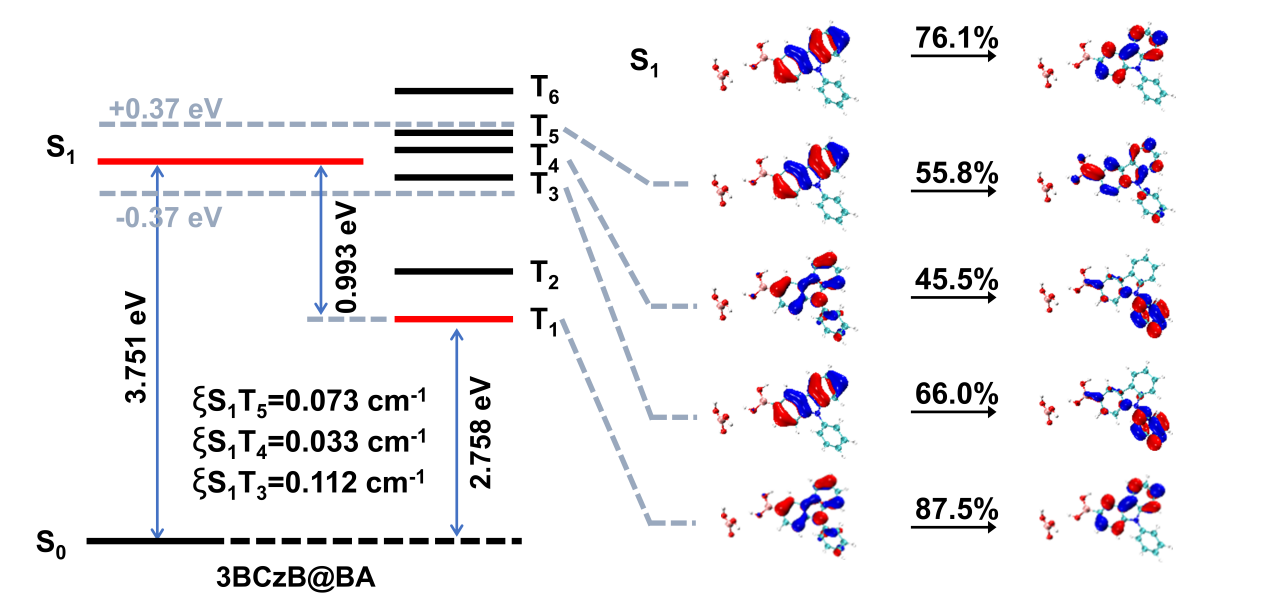


**Figure S25.** Calculated excitation energies, spin-orbit couplings (ξ) and natural transition orbitals for 3BCzB@BA.


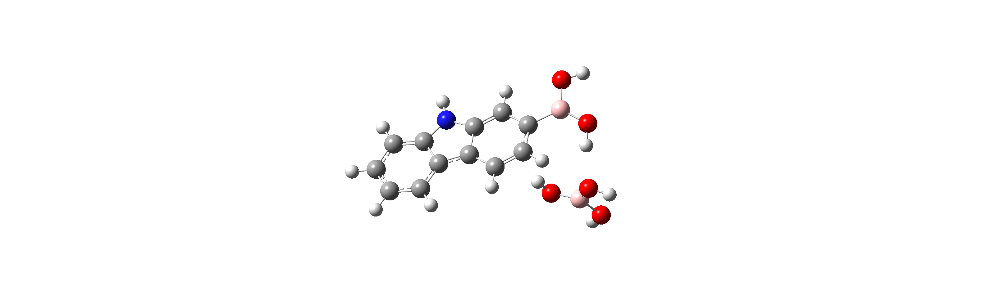


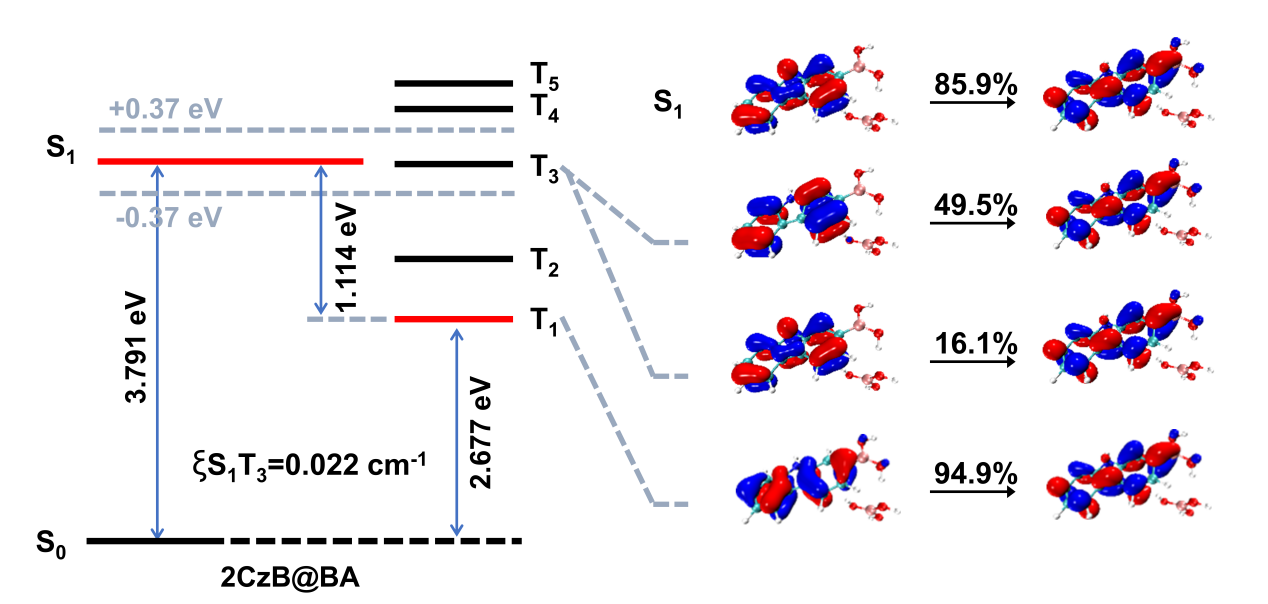


**Figure S26.** Calculated excitation energies, spin-orbit couplings (ξ) and natural transition orbitals for 2CzB@BA.


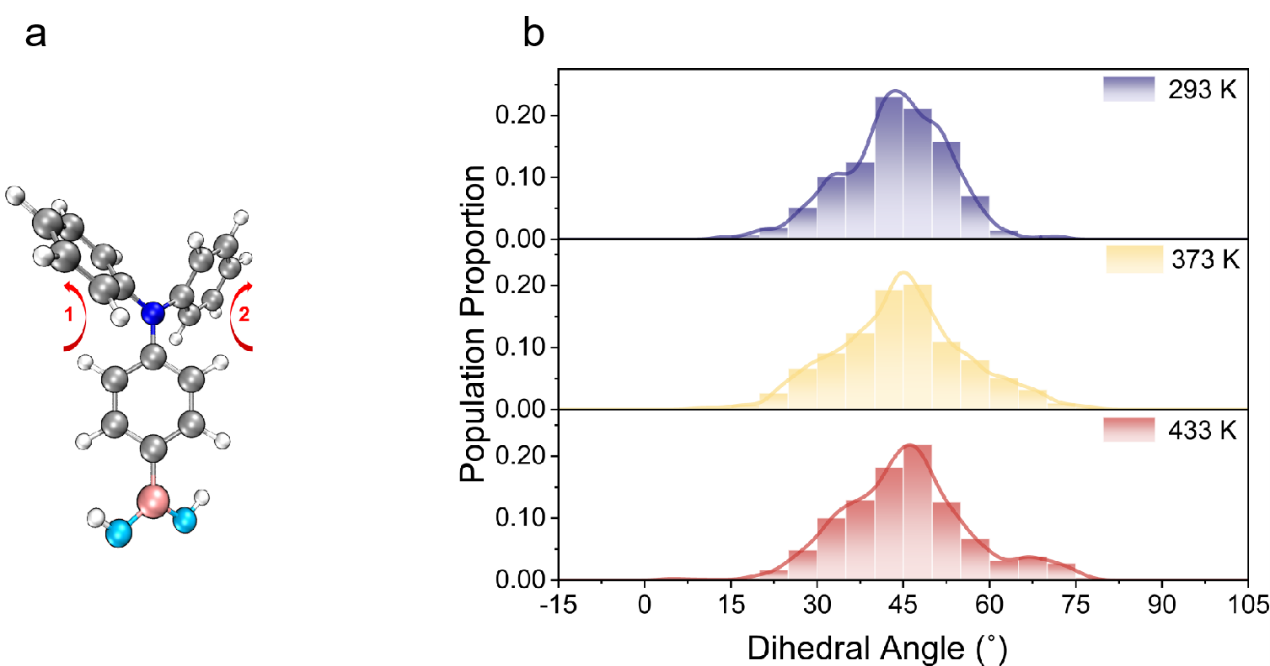


**Figure S27.** a) Torsion angles of guest TPBA. b) Distribution of the torsion angle at position 2 of TPBA at various temperatures.


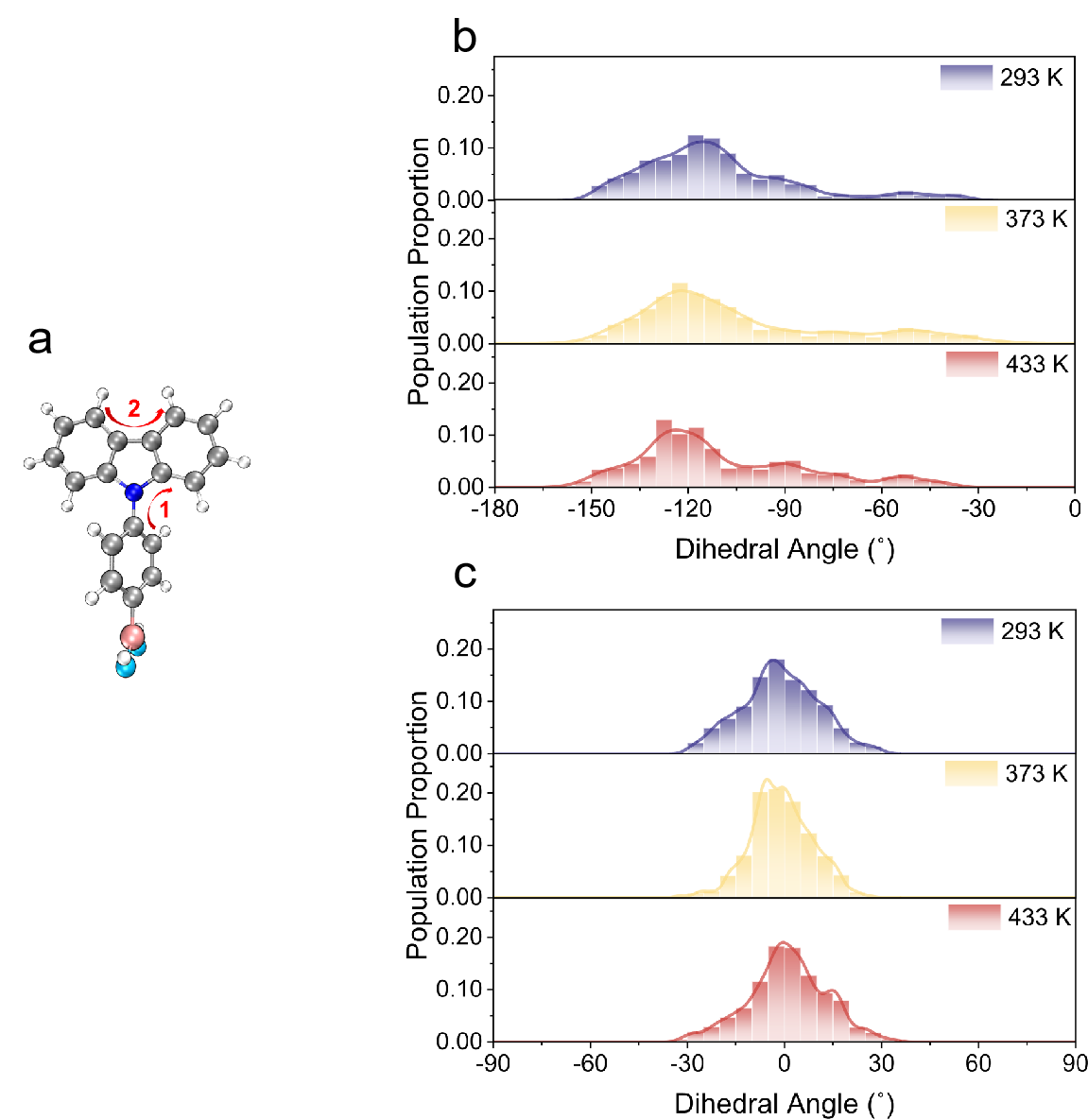


**Figure S28.** a) Torsion angles of guest 4BCzB. b) Distribution of the torsion angle at position 1 of 4BCzB at various temperatures. c) Distribution of the torsion angle at position 2 of 4BCzB at various temperatures.


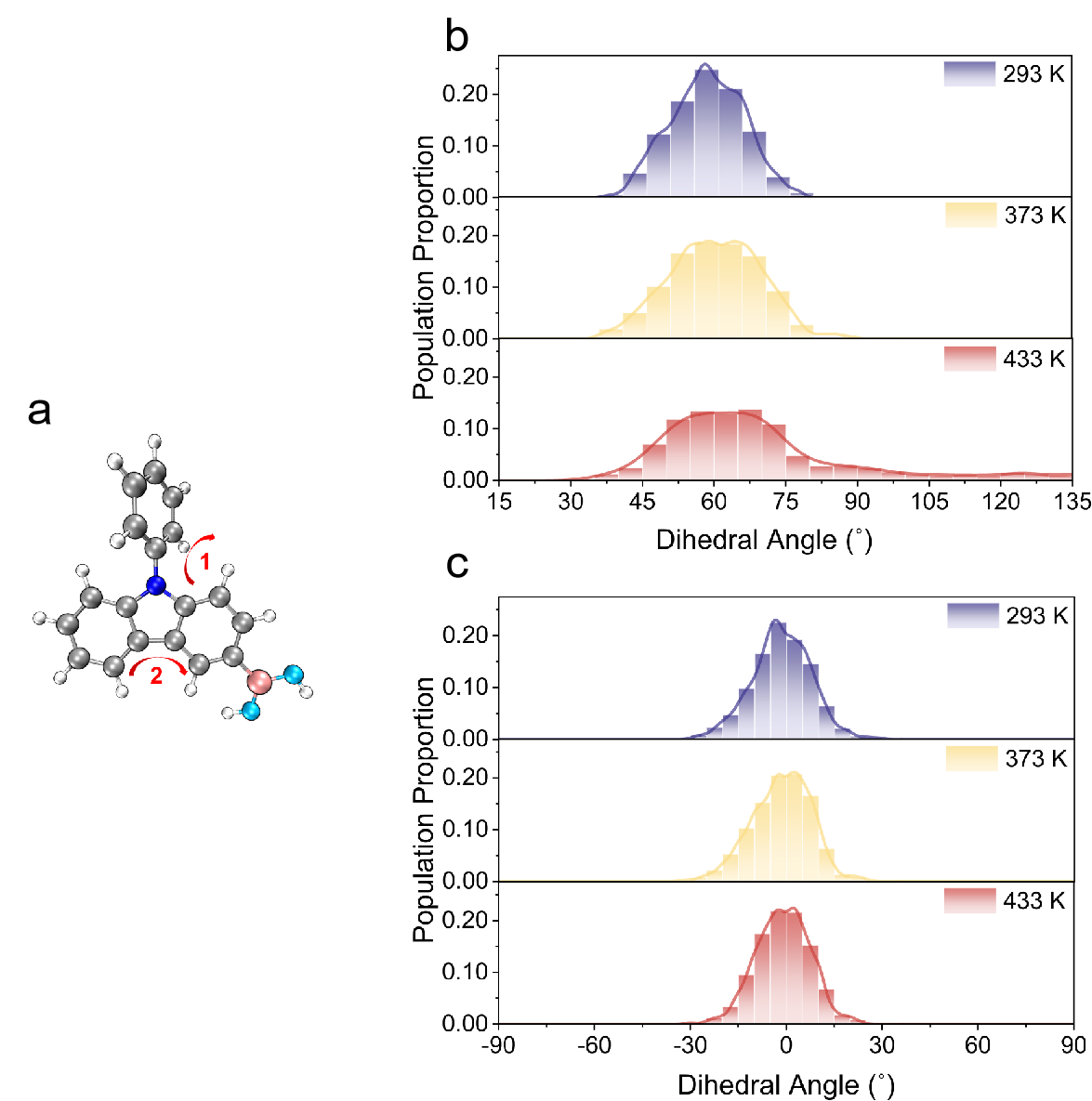


**Figure S29.** a) Torsion angles of guest 3BCzB. b) Distribution of the torsion angle at position 1 of 3BCzB at various temperatures. c) Distribution of the torsion angle at position 2 of 3BCzB at various temperatures.


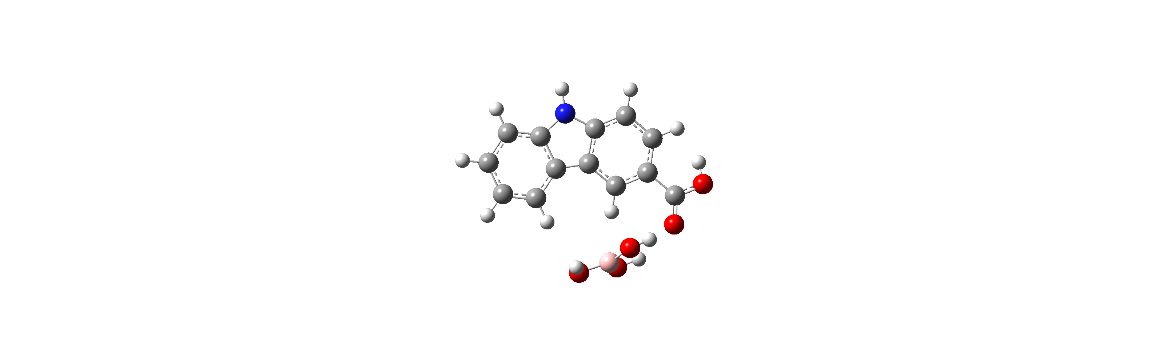


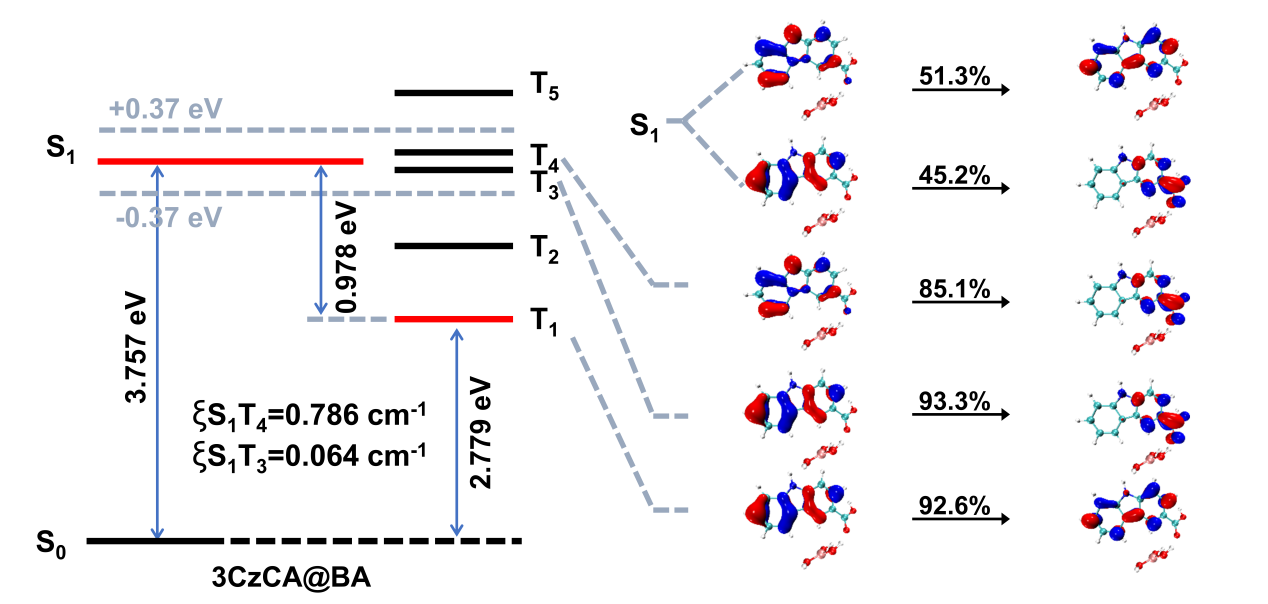


**Figure S30.** Calculated excitation energies, spin-orbit couplings (ξ) and natural transition orbitals for 3CzCA@BA.


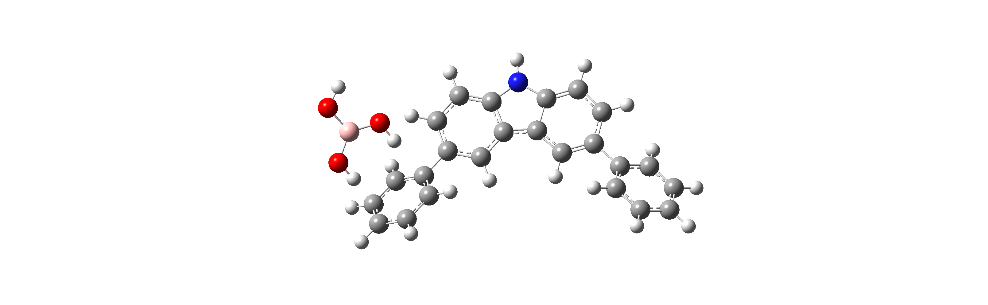


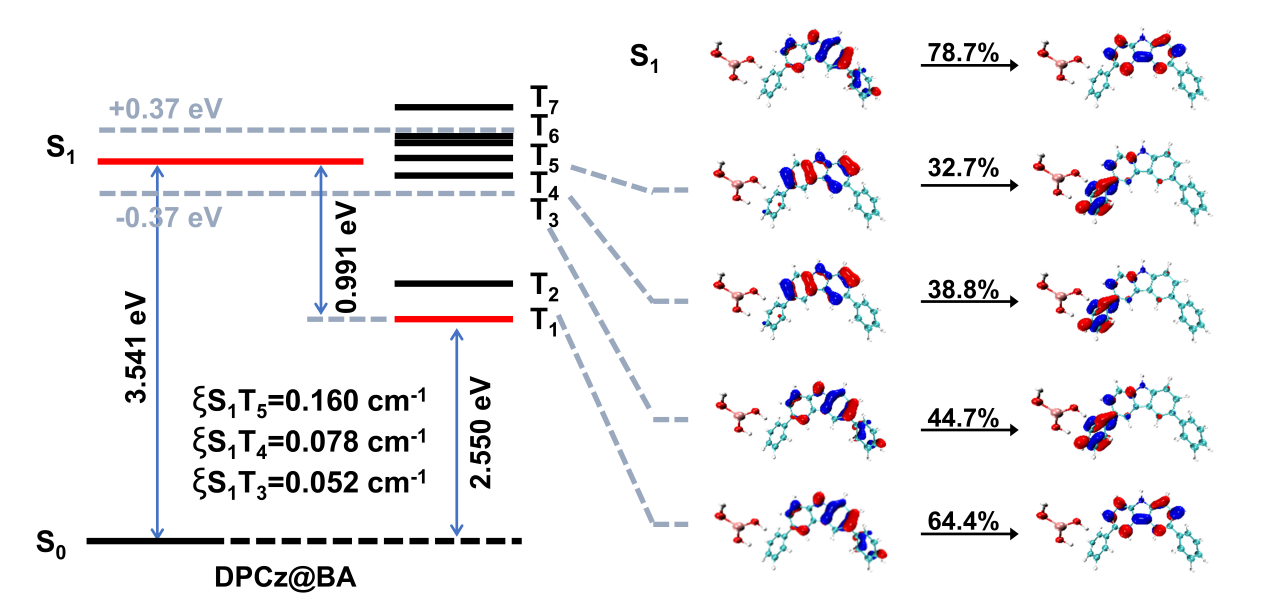


**Figure S31.** Calculated excitation energies, spin-orbit couplings (ξ) and natural transition orbitals for DPCz@BA.


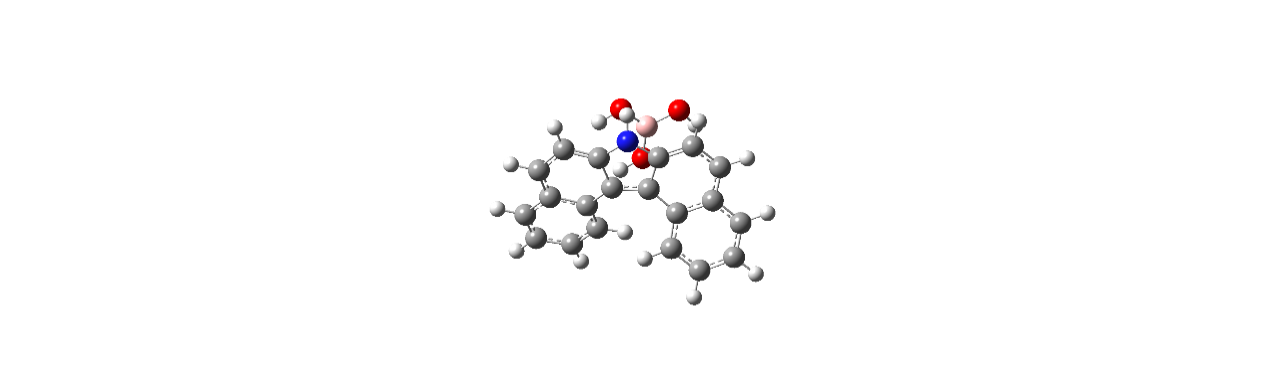


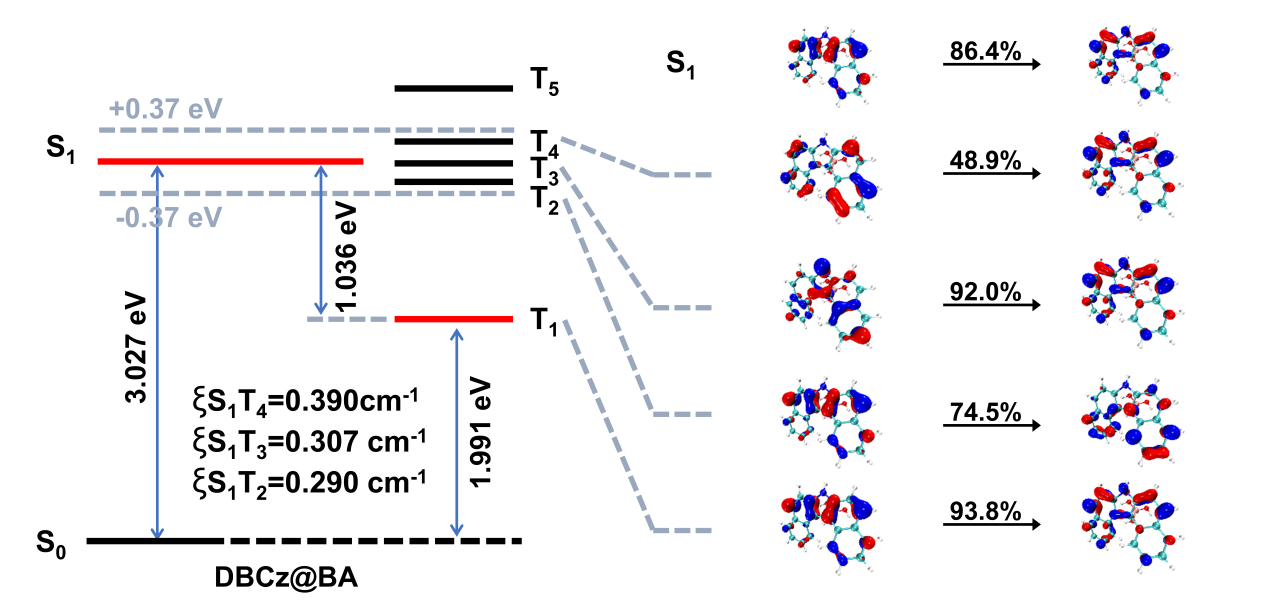


**Figure S32.** Calculated excitation energies, spin-orbit couplings (ξ) and natural transition orbitals for DBCz@BA.

**Table S5.** The singlet-triplet splitting energies (*E*_S1_*-E*_Tn_) and SOC constants from S_1_ to T_n_ of TPBA@BA, 4BCzB@BA, 3BCzB@BA, 2CzB@BA, 3CzCA@BA, DPCz@BA and DBCz@BA. The efficient intersystem crossing channels with │*E*_S1_*-E*_Tn_│ < 0.37 eV were highlighted in red.

| **Molecule** | **Transition** | ***E*_S1_-*E*_Tn_ (eV)** | **SOC (cm^-1^)** |
| --- | --- | --- | --- |
| TPBA@BA | *S_1_→T_1_* | 0.785 | 0.414 |
|  | *S_1_→T_2_* | -0.117 | 1.217 |
|  | *S_1_→T_3_* | -0.231 | 0.971 |
|  | *S_1_→T_4_* | -0.602 | 0.734 |
|  | *S_1_→T_5_* | -0.72 | 0.341 |
| 4BCzB@BA | *S_1_→T_1_* | 0.951 | 0.126 |
|  | *S_1_→T_2_* | 0.518 | 0.047 |
|  | *S_1_→T_3_* | 0.373 | 0.163 |
|  | *S_1_→T_4_* | 0.078 | 0.072 |
|  | *S_1_→T_5_* | -0.184 | 0.082 |
|  | *S_1_→T_6_* | -0.438 | 1.308 |
| 3BCzB@BA | *S_1_→T_1_* | 0.993 | 0.140 |
|  | *S_1_→T_2_* | 0.605 | 0.114 |
|  | *S_1_→T_3_* | 0.047 | 0.112 |
|  | *S_1_→T_4_* | -0.120 | 0.033 |
|  | *S_1_→T_5_* | -0.226 | 0.073 |
|  | *S_1_→T_6_* | -0.372 | 0.487 |
| 2CzB@BA | *S_1_→T_1_* | 1.114 | 0.209 |
|  | *S_1_→T_2_* | 0.746 | 0.046 |
|  | *S_1_→T_3_* | -0.282 | 0.022 |
|  | *S_1_→T_4_* | -0.387 | 0.169 |
|  | *S_1_→T_5_* | -0.540 | 0.024 |
| 3CzCA@BA | *S_1_→T_1_* | 0.978 | 0.843 |
|  | *S_1_→T_2_* | 0.526 | 0.032 |
|  | *S_1_→T_3_* | 0.365 | 0.064 |
|  | *S_1_→T_4_* | 0.166 | 0.786 |
|  | *S_1_→T_5_* | -0.491 | 0.496 |
| DPCz@BA | *S_1_→T_1_* | 0.991 | 0.230 |
|  | *S_1_→T_2_* | 0.553 | 0.120 |
|  | *S_1_→T_3_* | 0.238 | 0.052 |
|  | *S_1_→T_4_* | 0.197 | 0.078 |
|  | *S_1_→T_5_* | -0.118 | 0.160 |
|  | *S_1_→T_6_* | -0.247 | 0.102 |
|  | *S_1_→T_7_* | -0.493 | 0.117 |
| DBCz@BA | *S_1_→T_1_* | 1.144 | 0.210 |
|  | *S_1_→T_2_* | 0.111 | 0.290 |
|  | *S_1_→T_3_* | -0.252 | 0.307 |
|  | *S_1_→T_4_* | -0.341 | 0.390 |
|  | *S_1_→T_5_* | -0.529 | 0.460 |

**Table S6.** The singlet and triplet excited states transition configurations of TPBA@BA revealed by TD-DFT calculations. The efficient intersystem crossing channels with │*E*_S1_*-E*_Tn_│ < 0.37 eV were highlighted in red.

|  | **n** | **Energy (eV)** | **Orbitals** | **Transition** |
| --- | --- | --- | --- | --- |
| *S_n_* | *S_1_* | 3.120 | H→L | 0.953 |
| *T_n_* | *T_1_* | 2.335 | H→L | 0.922 |
|  | *T_2_* | 3.237 | H→L+2 | 0.786 |
|  |  |  | H→L+1 | 0.091 |
|  | *T_3_* | 3.351 | H→L+1 | 0.869 |
|  |  |  | H→L+2 | 0.087 |
|  | *T_4_* | 3.722 | H-3→L | 0.313 |
|  |  |  | H-1→L | 0.201 |
|  |  |  | H→L+5 | 0.122 |
|  |  |  | H→L+3 | 0.056 |
|  | *T_5_* | 3.840 | H→L+3 | 0.836 |

**Table S7.** The singlet and triplet excited states transition configurations of 4BCzB@BA revealed by TD-DFT calculations. The efficient intersystem crossing channels with │*E*_S1_*-E*_Tn_│ < 0.37 eV were highlighted in red.

|  | **n** | **Energy (eV)** | **Orbitals** | **Transition** |
| --- | --- | --- | --- | --- |
| *S_n_* | *S_1_* | 3.686 | H→L | 0.646 |
|  |  |  | H→L+1 | 0.345 |
| *T_n_* | *T_1_* | 2.735 | H→L+1 | 0.500 |
|  |  |  | H→L | 0.444 |
|  | *T_2_* | 3.168 | H-1→L | 0.502 |
|  |  |  | H-1→L+1 | 0.455 |
|  | *T_3_* | 3.313 | H-1→L+1 | 0.455 |
|  |  |  | H-1→L | 0.406 |
|  |  |  | H-3→L | 0.064 |
|  | *T_4_* | 3.608 | H→L | 0.511 |
|  |  |  | H→L+1 | 0.454 |
|  | *T_5_* | 3.870 | H-1→L+2 | 0.912 |
|  |  |  | H-3→L+2 | 0.052 |
|  | *T_6_* | 4.124 | H→L+2 | 0.834 |
|  |  |  | H→L | 0.066 |

**Table S8.** The singlet and triplet excited states transition configurations of 3BCzB@BA revealed by TD-DFT calculations. The efficient intersystem crossing channels with │*E*_S1_*-E*_Tn_│ < 0.37 eV were highlighted in red.

|  | **n** | **Energy (eV)** | **Orbitals** | **Transition** |
| --- | --- | --- | --- | --- |
| *S_n_* | *S_1_* | 3.751 | H-1→L | 0.761 |
|  |  |  | H→L | 0.098 |
|  |  |  | H→L+3 | 0.085 |
| *T_n_* | *T_1_* | 2.758 | H→L | 0.875 |
|  |  |  | H-1→L | 0.062 |
|  | *T_2_* | 3.146 | H-1→L | 0.889 |
|  |  |  | H→L | 0.064 |
|  | *T_3_* | 3.704 | H-1→L+1 | 0.660 |
|  |  |  | H→L+1 | 0.108 |
|  |  |  | H-3→L+1 | 0.093 |
|  | *T_4_* | 3.871 | H→L+1 | 0.455 |
|  |  |  | H→L+3 | 0.372 |
|  |  |  | H-1→L+1 | 0.007 |
|  | *T_5_* | 3.977 | H-1→L+3 | 0.558 |
|  |  |  | H-2→L | 0.117 |
|  |  |  | H→L+3 | 0.007 |
|  | *T_6_* | 4.123 | H-1→L+2 | 0.709 |
|  |  |  | H→L+2 | 0.130 |
|  |  |  | H-3→L+2 | 0.090 |

**Table S9.** The singlet and triplet excited states transition configurations of 2CzB@BA revealed by TD-DFT calculations. The efficient intersystem crossing channels with │*E*_S1_*-E*_Tn_│ < 0.37 eV were highlighted in red.

|  | **n** | **Energy (eV)** | **Orbitals** | **Transition** |
| --- | --- | --- | --- | --- |
| *S_n_* | *S_1_* | 3.791 | H-1→L | 0.859 |
|  |  |  | H→L+1 | 0.107 |
| *T_n_* | *T_1_* | 2.677 | H→L | 0.949 |
|  | *T_2_* | 3.045 | H-1→L | 0.967 |
|  | *T_3_* | 4.073 | H-2→L | 0.495 |
|  |  |  | H-1→L+2 | 0.161 |
|  |  |  | H-1→L+1 | 0.120 |
|  |  |  | H→L+2 | 0.093 |
|  | *T_4_* | 4.178 | H-3→L | 0.365 |
|  |  |  | H→L+2 | 0.190 |
|  |  |  | H→L+4 | 0.096 |
|  |  |  | H-2→L | 0.080 |
|  |  |  | H-1→L+1 | 0.076 |
|  |  |  | H→L+1 | 0.074 |
|  | *T_5_* | 4.331 | H→L+1 | 0.864 |
|  |  |  | H→L+2 | 0.053 |

**Table S10.** The singlet and triplet excited states transition configurations of 3CzCA@BA revealed by TD-DFT calculations. The efficient intersystem crossing channels with │*E*_S1_*-E*_Tn_│ < 0.37 eV were highlighted in red.

|  | **n** | **Energy (eV)** | **Orbitals** | **Transition** |
| --- | --- | --- | --- | --- |
| *S_n_* | *S_1_* | 3.757 | H-1→L | 0.513 |
|  |  |  | H→L+1 | 0.452 |
| *T_n_* | *T_1_* | 2.779 | H→L | 0.926 |
|  | *T_2_* | 3.231 | H-1→L | 0.949 |
|  | *T_3_* | 3.392 | H→L+1 | 0.933 |
|  | *T_4_* | 3.591 | H-1→L+1 | 0.851 |
|  |  |  | H-2→L+1 | 0.092 |
|  | *T_5_* | 4.248 | H-2→L | 0.477 |
|  |  |  | H-1→L+2 | 0.268 |
|  |  |  | H→L+2 | 0.126 |

**Table S11.** The singlet and triplet excited states transition configurations of DPCz@BA revealed by TD-DFT calculations. The efficient intersystem crossing channels with │*E*_S1_*-E*_Tn_│ < 0.37 eV were highlighted in red.

|  | **n** | **Energy (eV)** | **Orbitals** | **Transition** |
| --- | --- | --- | --- | --- |
| *S_n_* | *S_1_* | 3.541 | H→L | 0.787 |
|  |  |  | H-1→L | 0.100 |
| *T_n_* | *T_1_* | 2.550 | H→L | 0.644 |
|  |  |  | H-1→L | 0.225 |
|  |  |  | H-2→L | 0.051 |
|  | *T_2_* | 2.988 | H-1→L | 0.623 |
|  |  |  | H→L | 0.274 |
|  | *T_3_* | 3.303 | H→L+1 | 0.447 |
|  |  |  | H→L+3 | 0.243 |
|  |  |  | H-1→L | 0.071 |
|  |  |  | H-2→L+3 | 0.066 |
|  | *T_4_* | 3.344 | H-1→L+1 | 0.388 |
|  |  |  | H→L+3 | 0.293 |
|  |  |  | H-2→L+1 | 0.095 |
|  | *T_5_* | 3.659 | H-1→L+1 | 0.327 |
|  |  |  | H→L+1 | 0.303 |
|  |  |  | H→L+3 | 0.156 |
|  | *T_6_* | 3.788 | H-2→L | 0.376 |
|  |  |  | H-1→L+3 | 0.098 |
|  |  |  | H-6→L | 0.084 |
|  |  |  | H→L+5 | 0.068 |
|  | *T_7_* | 4.034 | H-3→L+4 | 0.193 |
|  |  |  | H-1→L+1 | 0.110 |
|  |  |  | H-5→L+2 | 0.102 |

**Table S12.** The singlet and triplet excited states transition configurations of DBCz@BA revealed by TD-DFT calculations. The efficient intersystem crossing channels with │*E*_S1_*-E*_Tn_│ < 0.37 eV were highlighted in red.

|  | **n** | **Energy (eV)** | **Orbitals** | **Transition** |
| --- | --- | --- | --- | --- |
| *S_n_* | *S_1_* | 3.189 | H→L | 0.864 |
| *T_n_* | *T_1_* | 2.045 | H→L | 0.938 |
|  | *T_2_* | 3.078 | H→L+1 | 0.745 |
|  |  |  | H-2→L | 0.177 |
|  | *T_3_* | 3.441 | H-1→L | 0.920 |
|  | *T_4_* | 3.530 | H-2→L | 0.489 |
|  |  |  | H→L+2 | 0.158 |
|  |  |  | H→L+1 | 0.157 |
|  |  |  | H-1→L+1 | 0.055 |
|  | *T_5_* | 3.718 | H-1→L+1 | 0.298 |
|  |  |  | H→L+2 | 0.234 |
|  |  |  | H-2→L+1 | 0.197 |
|  |  |  | H→L+3 | 0.088 |


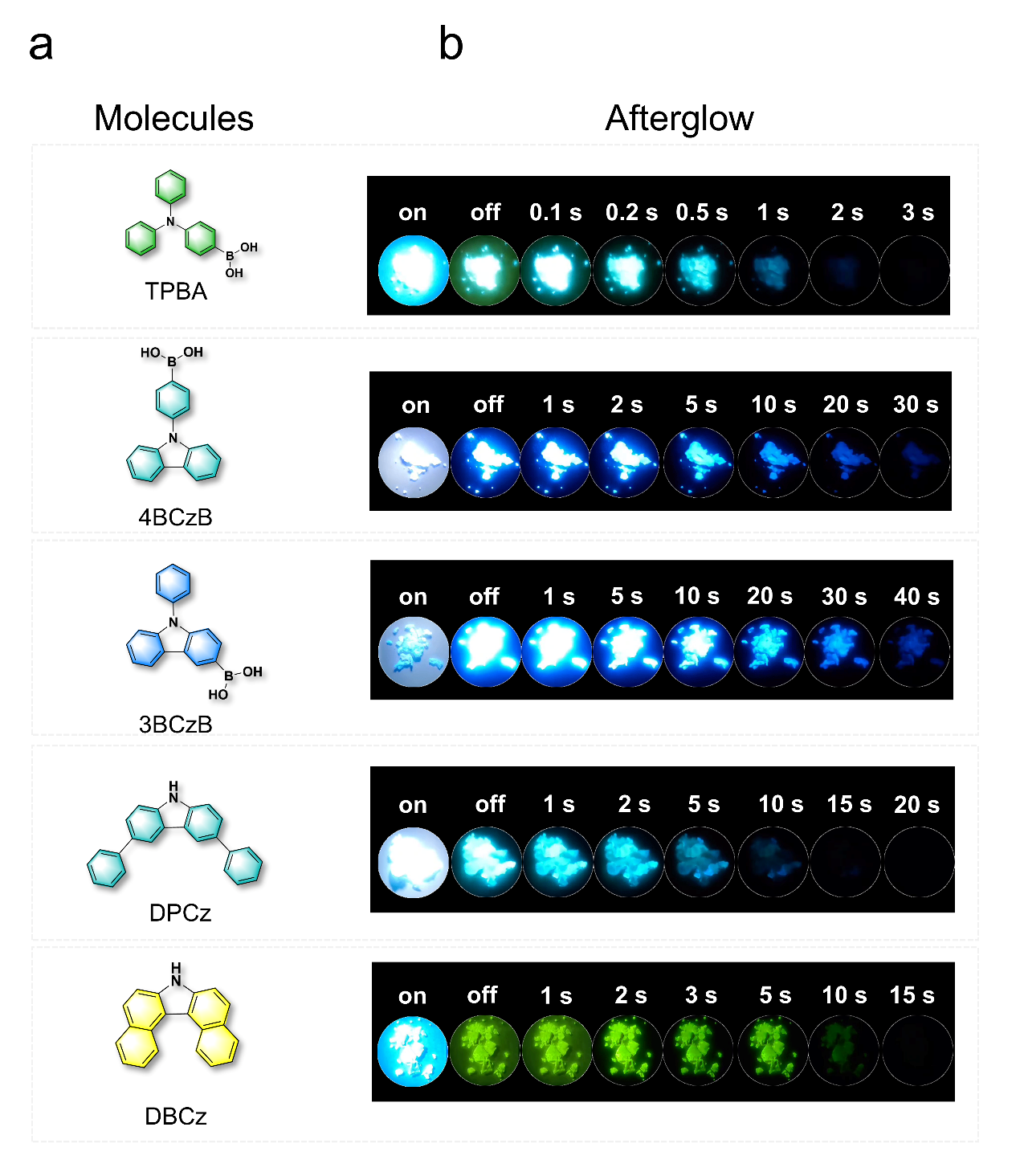


**Figure S33.** a) The structures of the guest molecules. b) The afterglow photographs of the prepared URTP materials


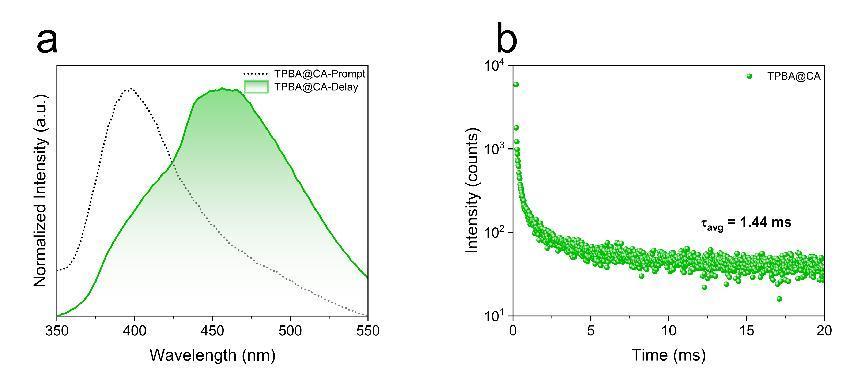


**Figure S34.** The prompt and delay emission spectra of TPBA@CA excited by 310 nm (delay time 10 ms). b) The phosphorescent lifetime of TPBA@CA excited by 310 nm.


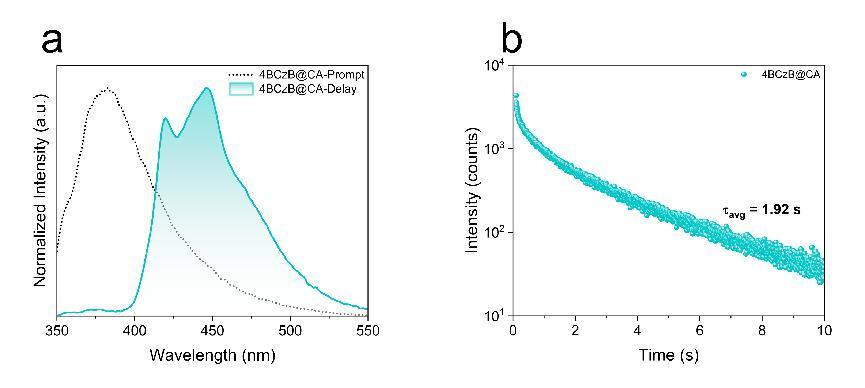


**Figure S35.** The prompt and delay emission spectra of 4BCzB@CA excited by 310 nm (delay time 10 ms). b) The phosphorescent lifetime of 4BCzB@CA excited by 310 nm.


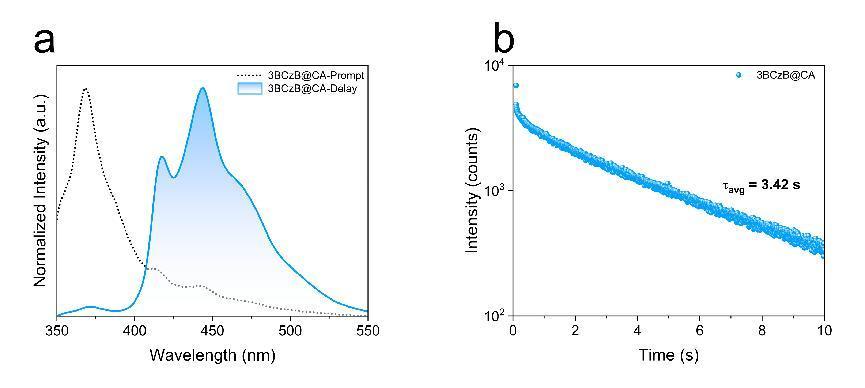


**Figure S36.** The prompt and delay emission spectra of 3BCzB@CA excited by 310 nm (delay time 10 ms). b) The phosphorescent lifetime of 3BCzB@CA excited by 310 nm.


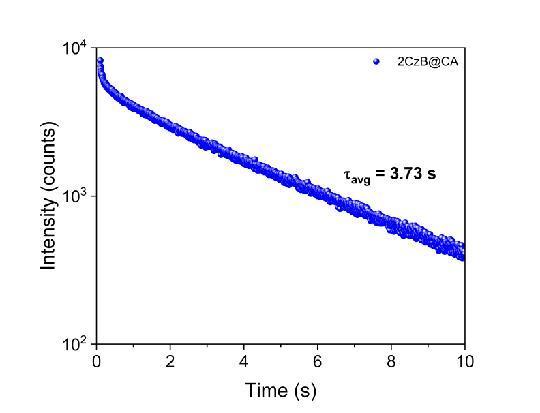


**Figure S37.** The phosphorescent lifetime of 2CzB@CA excited by 310 nm.


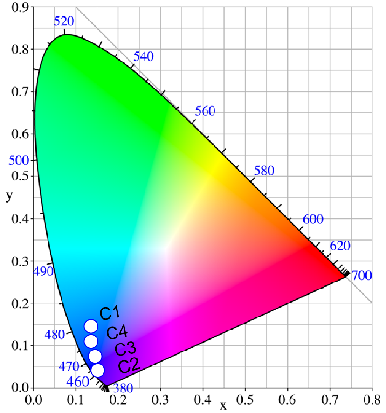


**Figure S38.** Chromaticity coordinates (x, y) calculated from the phosphorescence spectra of C1: TPBA@CA (0.14, 0.15), C2: 4BCzB@CA (0.15, 0.05), C3: 3BCzB@CA (0.15, 0.07), and C4: 2CzB@CA (0.14, 0.11).


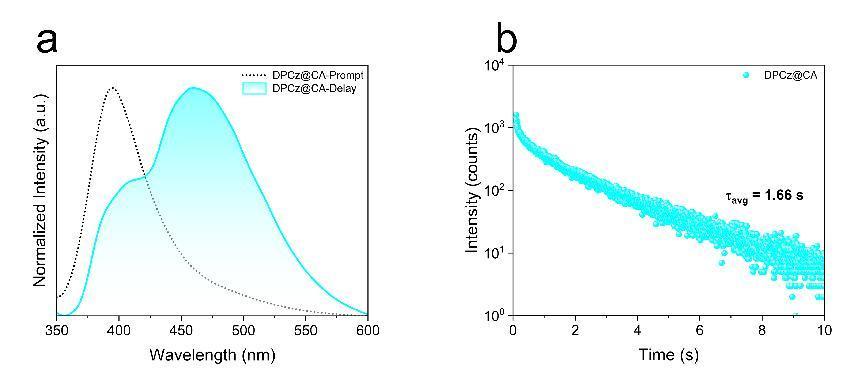


**Figure S39.** The prompt and delay emission spectra of DPCz@CA excited by 310 nm (delay time 10 ms). b) The phosphorescent lifetime of DPCz@CA excited by 310 nm.


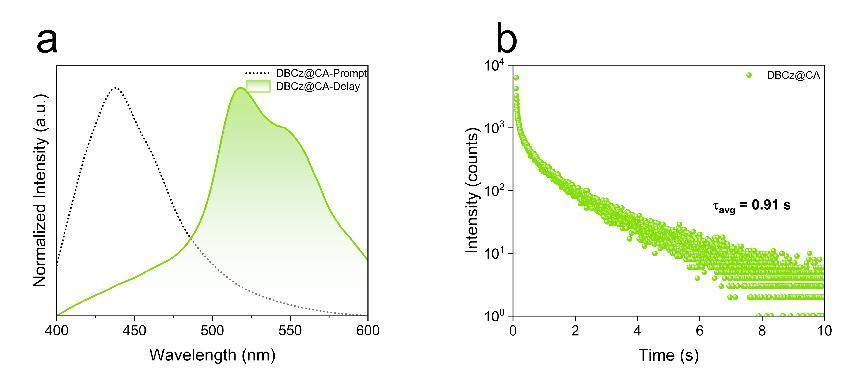


**Figure S40.** The prompt and delay emission spectra of DBCz@CA excited by 360 nm (delay time 10 ms). b) The phosphorescent lifetime of DBCz@CA excited by 360 nm.


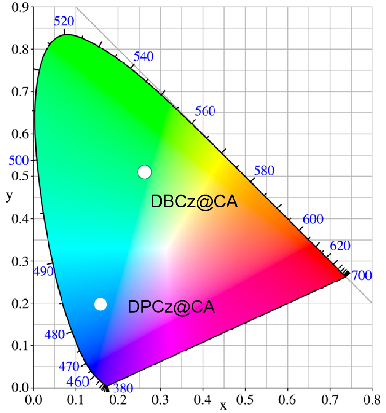


**Figure S41.** Chromaticity coordinates (x, y) calculated from the phosphorescence spectra of DPCz@CA (0.16, 0.19) and DBCz@CA (0.26, 0.50).


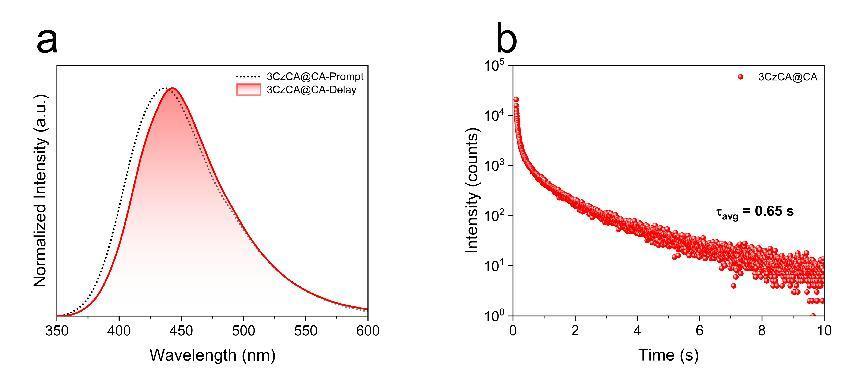


**Figure S42.** The prompt and delay emission spectra of 3CzCA@CA excited by 310 nm (delay time 10 ms). b) The phosphorescent lifetime of 3CzCA@CA excited by 310 nm.


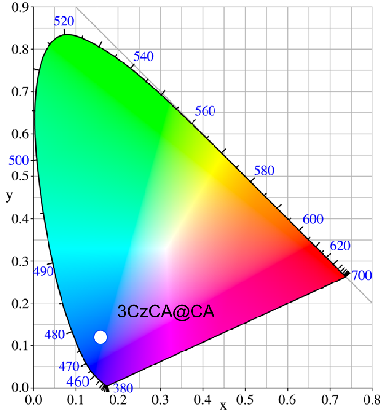


**Figure S43.** Chromaticity coordinates (x, y) calculated from the phosphorescence spectra of 3CzCA@CA (0.16, 0.12).

**Table S13.** The excited wavelength (Ex), photoluminescence wavelength (PL), phosphorescent wavelength (Phos), phosphorescent lifetime (τ_avg_), FLQY (fluorescence quantum yield), PhQY (phosphorescent quantum yield), and PhQY/PLQY of TPBA@CA, 4BCzB@CA, 3BCzB@CA, 2CzB@CA, DPCz@CA, DBCz@CA, and 3CzCA@CA.

|  | Ex (nm) | PL (nm) | Phos (nm) | τ_avg_ (s) | FLQY | PhQY | PhQY/PLQY |
| --- | --- | --- | --- | --- | --- | --- | --- |
| TPBA@CA | 310 | 397 | 457 | 0.00144 | 16.2% | 13.8% | 46.1% |
| 4BCzB@CA | 310 | 382 | 446 | 1.92 | 24.0% | 12.0% | 49.8% |
| 3BCzB@CA | 310 | 366 | 443 | 3.42 | 13.9% | 4.1% | 22.8% |
| 2CzB@CA | 310 | 388 | 442 | 3.73 | 54.2% | 32.8% | 37.7% |
| DPCz@CA | 310 | 402 | 450 | 1.66 | 22.2% | 8.8% | 28.3% |
| DBCz@CA | 360 | 438 | 518 | 0.91 | 17.9% | 3.1% | 14.6% |
| 3CzCA@CA | 310 | 437 | 442 | 0.65 | 0.05% | 44.5% | 98.9% |

**Table S14.** Comparison of this work with the reported properties of phosphorescent powders. Where the factor of quality (FQ) = phosphorescence lifetime (τ_avg_) * phosphorescence quantum yield (PhQY)^[3]^.

| Sample | Name | τ_avg_ (s) | PhQY | FQ |
| --- | --- | --- | --- | --- |
| 1 | CT5-20^[2]^ | 1.67 | 46.1% | 0.77 |
| 2 | MCATMA^[3]^ | 1.30 | 65.0% | 0.84 |
| 3 | P2BA@U_60_^[4]^ | 5.33 | 10.82% | 0.58 |
| 4 | TSP^[5]^ | 0.168 | 96.5% | 0.16 |
| 5 | EPBA^[6]^ | 1.85 | 53.0% | 0.98 |
| 6 | Asp-CDs/BA^[7]^ | 0.53 | 54.27% | 0.29 |
| 7 | a-CD_S_/BA^[8]^ | 1.60 | 8.7% | 0.14 |
| 8 | BA@PHA^[9]^ | 2.01 | 32.0% | 0.64 |
| 9 | PA@BA^[10]^ | 1.06 | 11.49% | 0.12 |
| 10 | 2-CA^[11]^ | 0.80 | 76.42% | 0.61 |
| **11** | **2CzB@BA(This Work)** | **5.44** | **12.2%** | **0.66** |
| **12** | **2CzB@CA(This Work)** | **3.73** | **32.8%** | **1.22** |


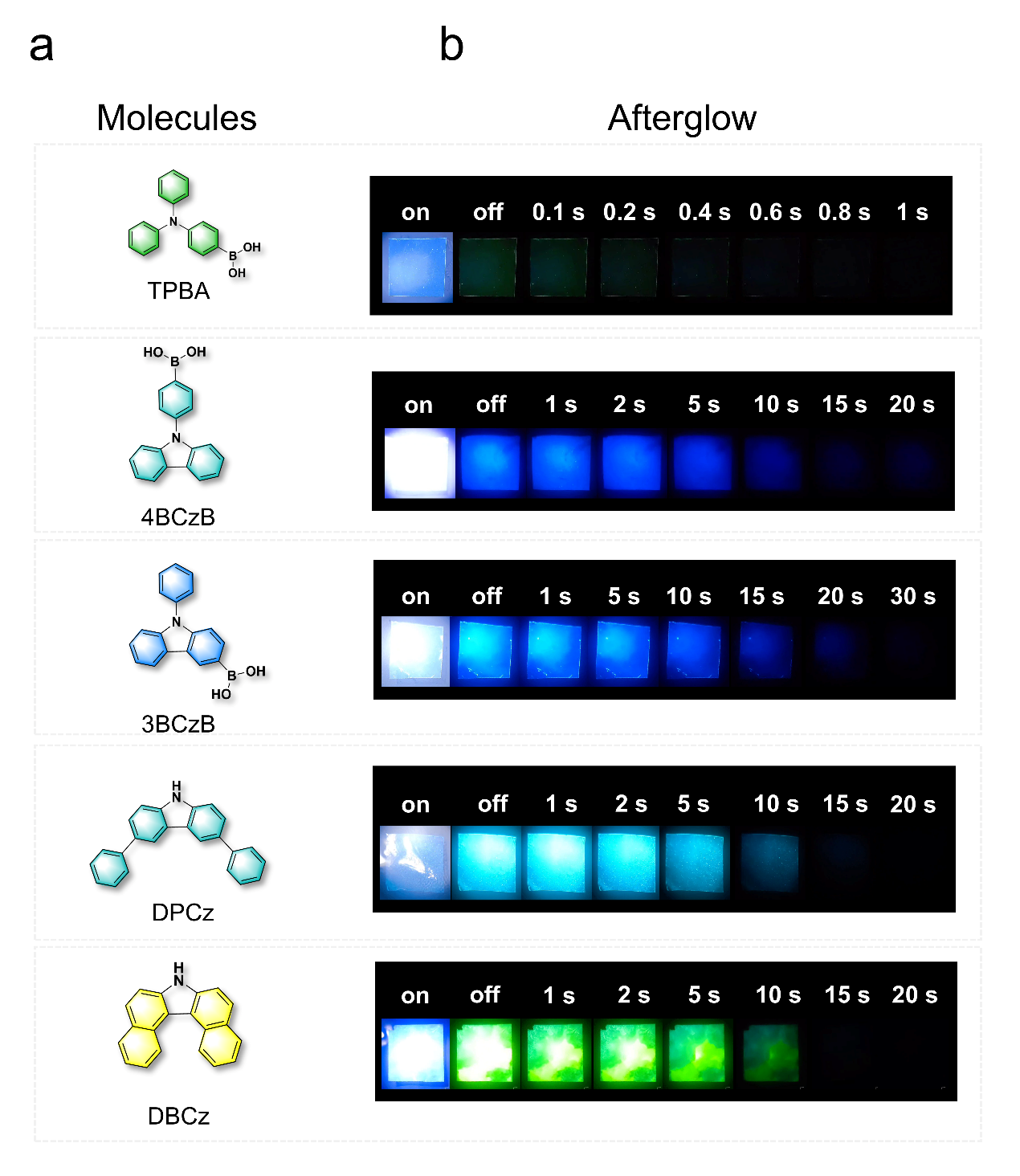


**Figure S44.** a) The structures of the guest molecules. b) The afterglow photographs of the prepared URTP materials.


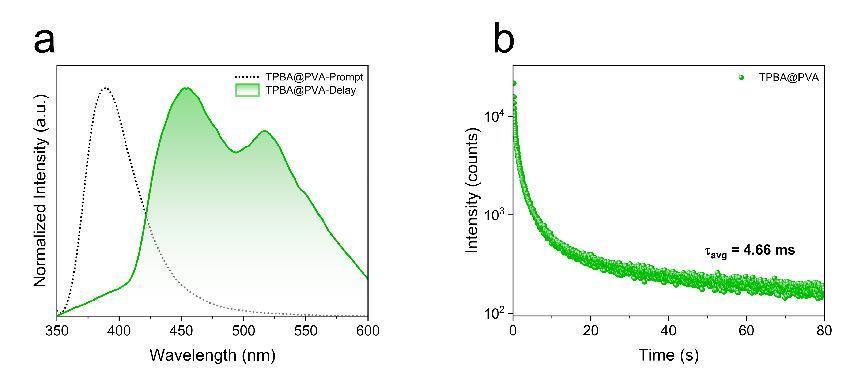


**Figure S45.** The prompt and delay emission spectra of TPBA@PVA excited by 310 nm (delay time 10 ms). b) The phosphorescent lifetime of TPBA@PVA excited by 310 nm.


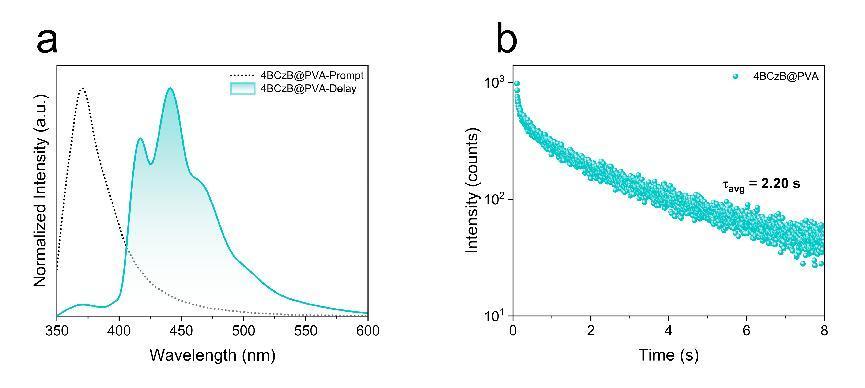


**Figure S46.** The prompt and delay emission spectra of 4BCzB@PVA excited by 310 nm (delay time 10 ms). b) The phosphorescent lifetime of 4BCzB@PVA excited by 310 nm.


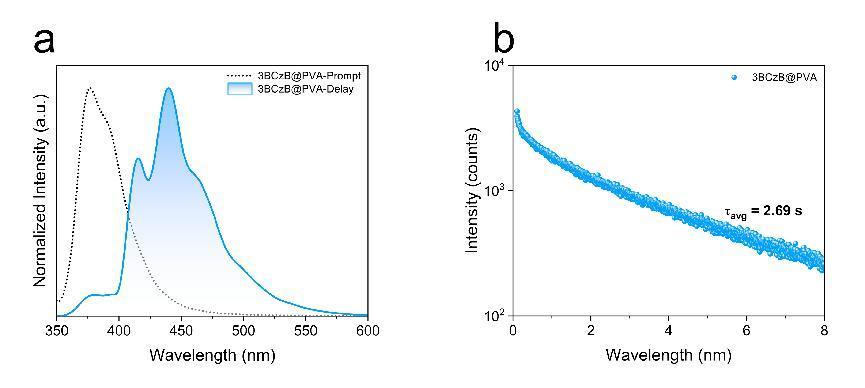


**Figure S47.** The prompt and delay emission spectra of 3BCzB@PVA excited by 310 nm (delay time 10 ms). b) The phosphorescent lifetime of 3BCzB@PVA excited by 310 nm.


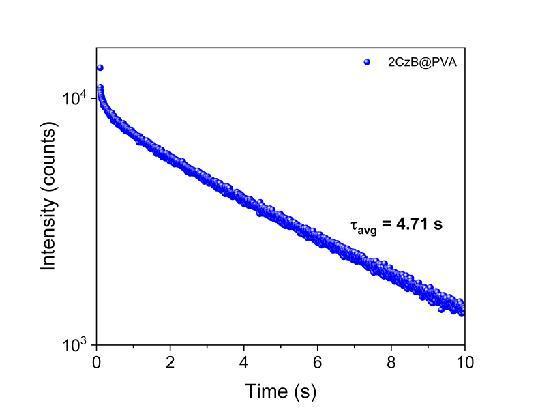


**Figure S48.** The phosphorescent lifetime of 2CzB@PVA excited by 310 nm.


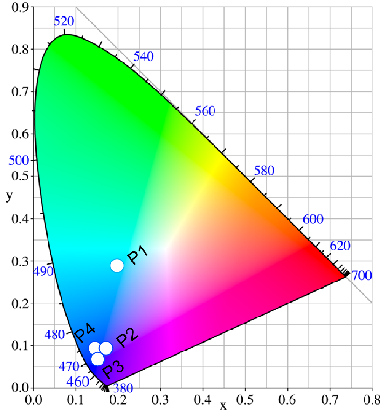


**Figure S49.** Chromaticity coordinates (x, y) calculated from the phosphorescence spectra of P1: TPBA@PVA (0.19, 0.28), P2: 4BCzB@PVA (0.16, 0.09), P3: 3BCzB@PVA (0.15, 0.08), and P4: 2CzB@PVA (0.15, 0.09).


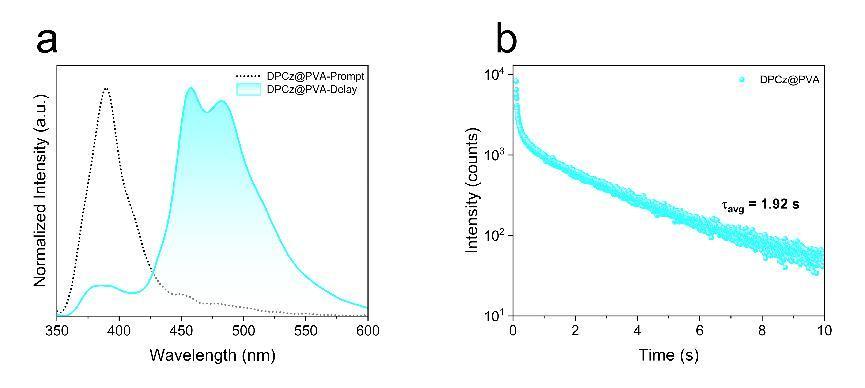


**Figure S50.** The prompt and delay emission spectra of DPCz@PVA excited by 310 nm (delay time 10 ms). b) The phosphorescent lifetime of DPCz@PVA excited by 310 nm.


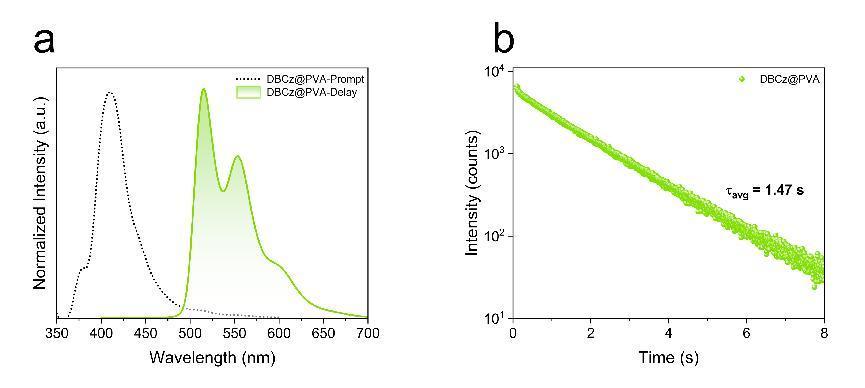


**Figure S51.** The prompt and delay emission spectra of DBCz@PVA excited by 360 nm (delay time 10 ms). b) The phosphorescent lifetime of DBCz@PVA excited by 360 nm.


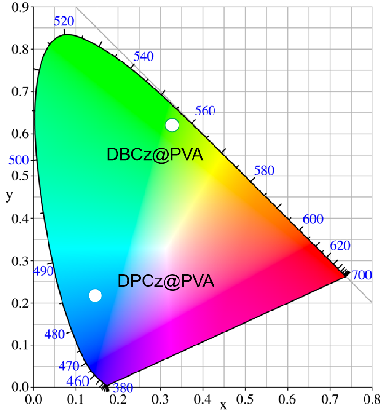


**Figure S52.** Chromaticity coordinates (x, y) calculated from the phosphorescence spectra of DPCz@PVA (0.15, 0.21) and DBCz@PVA (0.33, 0.62).

**Table S15.** The excited wavelength (Ex), photoluminescence wavelength (PL), phosphorescent wavelength (Phos), phosphorescent lifetime (τ_avg_), FLQY (fluorescence quantum yield), PhQY (phosphorescent quantum yield), and PhQY/PLQY of TPBA@CA, 4BCzB@CA, 3BCzB@CA, 2CzB@CA, DPCz@CA, and DBCz@CA.

|  | Ex (nm) | PL (nm) | Phos (nm) | τ_avg_ (s) | FLQY | PhQY | PhQY/PLQY |
| --- | --- | --- | --- | --- | --- | --- | --- |
| TPBA@PVA | 310 | 388 | 454 | 0.00466 | 18.2% | 2.8% | 13.3% |
| 4BCzB@PVA | 310 | 371 | 441 | 2.20 | 13.2% | 3.8% | 22.6% |
| 3BCzB@PVA | 310 | 377 | 440 | 2.69 | 21.8% | 7.2% | 24.8% |
| 2CzB@PVA | 310 | 381 | 430 | 4.71 | 21.8% | 6.2% | 22.0% |
| DPCz@PVA | 310 | 390 | 458 | 1.92 | 17.5% | 4.5% | 20.3% |
| DBCz@PVA | 360 | 410 | 515 | 1.47 | 19.1% | 1.9% | 9.2% |

**Reference**

[1] W. Ye, H. Ma, H. Shi, H. Wang, A. Lv, L. Bian, M. Zhang, C. Ma, K. Ling, M. Gu, Y. Mao, X. Yao, C. Gao, K. Shen, W. Jia, J. Zhi, S. Cai, Z. Song, J. Li, Y. Zhang, S. Lu, K. Liu, C. Dong, Q. Wang, Y. Zhou, W. Yao, Y. Zhang, H. Zhang, Z. Zhang, X. Hang, Z. An, X. Liu, W. Huang, *Nature Materials* **2021**, *20*, 1539.

[2] S. Xu, W. Wang, H. Li, J. Zhang, R. Chen, S. Wang, C. Zheng, G. Xing, C. Song, W. Huang, *Nature Communications* **2020**, *11*, 4802.

[3] J. Zhang, S. Xu, L. Zhang, X. Wang, Y. Bian, S. Tang, R. Zhang, Y. Tao, W. Huang, R. Chen, *Advanced Materials* **2022**, *34*, 2206712.

[4] Q. Jia, X. Yan, B. Wang, J. Li, W. Xu, Z. Shen, C. Bo, Y. Li, L. Chen, *Nature Communications* **2023**, *14*, 4164.

[5] W. Ye, H. Ma, H. Shi, H. Wang, A. Lv, L. Bian, M. Zhang, C. Ma, K. Ling, M. Gu, Y. Mao, X. Yao, C. Gao, K. Shen, W. Jia, J. Zhi, S. Cai, Z. Song, J. Li, Y. Zhang, S. Lu, K. Liu, C. Dong, Q. Wang, Y. Zhou, W. Yao, Y. Zhang, H. Zhang, Z. Zhang, X. Hang, Z. An, X. Liu, W. Huang, *Nature Materials* **2021**, *20*, 1539.

[6] Y. Huang, P. Li, *Chemical Engineering Journal* **2024**, *480*, 148157.

[7] Z. Li, S. Cao, Y. Zheng, L. Song, H. Zhang, Y. Zhao, *Advanced Functional Materials* **2024**, *34*, 2306956.

[8] W. Li, W. Zhou, Z. Zhou, H. Zhang, X. Zhang, J. Zhuang, Y. Liu, B. Lei, C. Hu, *Angewandte Chemie International Edition* **2019**, *58*, 7278.

[9] Z. Zhang, Z. Wang, X. Liu, Y. Shi, Z. Li, Y. Zhao, *Advanced Science* **2023**, *10*, 2300139.

[10] Z. Guan, Z. Tang, J. Zeng, J. Deng, Y. Zheng, H. Li, X. Liu, *Advanced Optical Materials* **2024**, *n/a*, 2302820.

[11] Z. Guan, Z. Tang, J. Deng, Y. Zheng, H. Li, X. Liu, *Advanced Functional Materials* **2024**, *34*, 2310198.
